# A printed luminescent flier inspired by plant seeds for eco-friendly physical sensing

**DOI:** 10.1126/sciadv.adi8492

**Published:** 2023-11-15

**Authors:** Kliton Cikalleshi, Albenc Nexha, Thomas Kister, Marilena Ronzan, Alessio Mondini, Stefano Mariani, Tobias Kraus, Barbara Mazzolai

**Affiliations:** ^1^ Bioinspired Soft Robotics Laboratory, Istituto Italiano di Tecnologia, Genova, Italy.; ^2^ The Biorobotics Institute, Scuola Superiore Sant’Anna, 56025 Pontedera, Italy.; ^3^ INM-Leibniz Institute for New Materials, 66123 Saarbrücken, Germany.; ^4^ Saarland University, Colloid and Interface Chemistry, 66123, Saarbrücken, Germany.

## Abstract

Continuous and distributed monitoring of environmental parameters may pave the way for developing sustainable strategies to tackle climate challenges. State-of-the-art technologies, made with electronic systems, are often costly, heavy, and generate e-waste. Here, we propose a new generation of self-deployable, biocompatible, and luminescent artificial flying seeds for wireless, optical, and eco-friendly monitoring of environmental parameters (i.e., temperature). Inspired by natural *Acer campestre* plant seeds, we developed three-dimensional functional printed luminescent seed–like fliers, selecting polylactic acid as a biocompatible matrix and temperature as a physical parameter to be monitored. The artificial seeds mimic the aerodynamic and wind dispersal performance of the natural ones. The sensing properties are given by the integration of fluorescent lanthanide–doped particles, whose photoluminescence properties depend on temperature. The luminescent artificial flying seeds can be optically read from a distance using eye-safe near-infrared wavelengths, thus acting as a deployable sensor for distributed monitoring of topsoil environmental temperatures.

## Teaser

Biocompatible, printed luminescent seed-inspired fliers sense environmental parameters (i.e., temperature) via optical readouts.

## INTRODUCTION

Wireless sensor networks connect multiple dispersed sensors to monitor and record distributed environmental parameters such as temperature, humidity and pollutants in air, seawater, and soil (*1*). Despite being at the core of current technological developments due to their flexibility, temporal, and spatial acquisitions at multiple areas (*1*), currently available sensors are time-expensive for spatial distribution and resource intensive as they may require wires, power supply, and frequent servicing. New technologies that use nature-inspired passive motion to enable, for example, wind dispersal and distribution at microscopic (e.g., pollen and fungi) and macroscopic [e.g., insects and plant seeds (*2*, *3*)] scales, can simplify deployment, particularly when combined with biodegradable or biocompatible passive sensing (*4*).

Recently, sensor networks bioinspired from flying plant seeds such as dandelion (*5*), *Tristellateia australasiae* (*6*, *7*), and *Alsomitra macrocarpa* (*8*) have been reported, confirming the attractiveness of these solutions. Iyer *et al.* (*5*) reported a battery-free wireless electronic device, bioinspired from natural dandelion seeds, to measure temperature, humidity, light, pressure, magnetic fields, and acceleration. However, the reported wireless sensor networks (*5*) rely on electronics, which are often costly and heavy and generate e-waste (*5*, *6*, *9*). Roger’s group (*7*) reported the first environmentally degradable flier bioinspired from natural *T. australasiae* seeds for remote and colorimetric assessments of environmental parameters such as pH, heavy metal concentrations, and ultraviolet (UV) exposure.

Maple seeds (genus *Acer*), also known as samaras, could inspire the creation of a new generation of wind-dispersible sensors. These seeds are carried passively by the wind and are dispersed at large distances and in distinct areas (*10*). A samara is composed of a seed section (nut or pericarp) and a single wing section. After abscission from the tree, samara seeds start autorotating, causing a constant descent speed, contrary to the one expected on its mass, gravitational acceleration, drag, and friction forces (*10*–*13*). In this way, the probability of being dispersed by the wind and spread is highly enhanced. Samara seeds portray a fascinating example of morphological computation in nature as their morphology enables the passive autorotation mechanism of flight, i.e., no additional energy input comes from the seed (*14*). Samara seeds have been built with electronic-based and not biocompatible sensor networks for in situ atmospheric monitoring (*15*, *16*) and fire detection (*16*).

Recent breakthroughs have led to the development of biocompatible and biodegradable sensors which, after fulfilling their functions, break down into residues that are not dangerous for the environment. This approach avoids the dispersal of new e-waste in a natural environment and obviates the retrieval of distributed sensors (*17*–*19*).

Here, we propose the first functional three-dimensional (3D) printed (*20*, *21*), self-deployable, and biocompatible artificial seeds, inspired by samara seeds (i.e., *Acer campestre*), for low-cost and eco-friendly monitoring of a physical parameter (i.e., temperature, as a proof of concept) via optical readout (i.e., photoluminescent readout). Compared to other artificial fliers bioinspired by dandelion (thickness from 7.5 to 25 μm) (*3*), *T. australasiae* (thickness of 140 μm) (*5*), or *A. macrocarpa* (thickness of 30 μm) seeds (*8*), the *A. campestre* seed has a thick pericarp (3.5 ± 0.2 mm) (fig. S1). The thick pericarp enables high signal-to-noise ratio detection and analysis of the optical signal, as required for single-step functional integration of optical sensing using functional 3D printing.

We apply a biomimetic and bioengineering single-step approach for the fabrication of deployable and functional fliers. A fluorescent sensor material is directly shaped into a bioinspired structure using functional 3D printing methodology (table S1) (*20*, *21*). This approach considerably simplifies fabrication compared to the state of the art of seed-inspired fliers for distributed environmental sensing, usually based on a subsequent integration of the electronic components and organic dyes (table S1). This reduction in complexity can enable the use of functional 3D printed fliers where economical or technological limitations are relevant. The fluorescent lanthanide–doped materials that add the sensor functionality exhibit stable photoluminescence, without photobleaching or degradation, and can operate under harsh conditions (*22*). The photoluminescence of the particles is temperature dependent (*23*), which renders the seeds as noncontact thermal sensor for passive sensing of environmental temperature. Current environmental sensors are based on organic molecules that are suitable for pH or UV sensing (*7*). Regardless, in scenarios where the environmental sensors are to sustain long periods of time (ranging from months to years) in the sunlight, inorganic phosphors such as the lanthanide-doped particles reported here, with their inherent photostability, are preferable.

In the first step, we studied the morphology, structure, aerodynamics, and dispersion parameters of the natural samara seeds with the goal of identifying key principles relevant to the design and development of the artificial seeds with the same aerodynamic and dispersion capabilities (*14*). We used fused deposition modeling as 3D printing technology to produce artificial samara seeds with the same aerodynamic and wind-distribution features. We chose polylactic acid (PLA) (*24*) because this polymer combines good printability with thermal and mechanical stability (*25*) and is suitable for the embedding of fluorescent sensors. Bulk PLA is sufficiently transparent in the visible (Vis) and the near-infrared (NIR) range (with transmittances around 95% for film samples) (*25*). Furthermore, PLA is an environmentally friendly and biocompatible polymer derived from renewable resources, such as corn starch (*26*). It belongs to the family of aliphatic polyesters commonly made with α-hydroxy acids (i.e., lactic acid) (*26*). It is environmentally degraded by hydrolysis of the ester bond (*26*), and the reported biodegradation in landfills and soils ranges from 20 to 1000 μm/year depending on the environmental and macrobiotic conditions (*27*) and with a mineralization rate of 10% every 150 days (*28*).

To produce fluorescent artificial samara seeds (hereafter I-SeedSam), fluorescent filaments made of lanthanide-doped materials and the PLA polymer were generated via a melt processing technique, followed by 3D printing. The lanthanide-doped materials are nontoxic and exhibit the ability to convert low excitation photon energies into high photon energies covering a wide range of wavelengths, spanning from UV, Vis, and NIR (*29*). We selected erbium (Er^3+^) and ytterbium (Yb^3+^) in a hexagonal NaYF_4_ host. The refractive index of this host is 1.47 (*30*) and matches that of the PLA matrix of approximately 1.48 (*31*), which minimizes scattering. The sensitizer Yb^3+^ efficiently absorbs light from widely available laser sources at eye-safe NIR wavelengths. The activator Er^3+^ converts the energy into green light with two separate emission bands around 520 and 540 nm that are suitable for ratiometric luminescence temperature sensing (*29*).

Temperature sensing could be realized by the change of the characteristics of the photoluminescence [such as bandwidth, spectral shift, fluorescence intensity ratio (FIR), lifetime, and polarization] when exposed to different temperatures (*23*). Among these, the FIR technique is particularly reliable, as it is the least affected by light from the surrounding environment and requires only comparatively simple optical setups (*23*). We performed ratiometric sensing by comparing the temperature-dependent ratio of the two green emission bands and evaluated the performance of the I-SeedSam as a temperature sensor within the environmental range from 268 to 313 K (i.e., from −5.15° to 39.85°C). The performance of the physical sensor is accurate for applications as passive sensors for environmental monitoring.

## RESULTS

### Morphometric and aerodynamic characterizations of *A. campestre* seeds

**Fig. 1.**
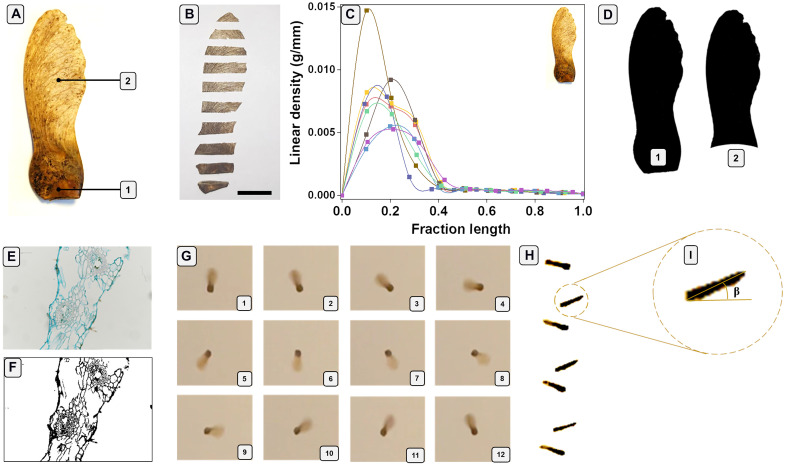
*A. campestre* seed morphometric and aerodynamic characterizations. (**A**) Picture of an *A. campestre* seed highlighting the main body parts: the nut or pericarp (as 1) and wing (as 2) section. (**B**) Seed sectioning. Scale bar, 1 cm. (**C**) Linear density (in grams per millimeter) of eight seeds after sectioning. (**D**) Image binarization of (A) with pericarp (1) and without pericarp (2) for the wing surface (*S*) and wing loading (*W*/*S*) estimation. (**E**) Histological analysis of the wing using Alcian blue for cellulose distribution estimation. (**F**) Binarization of (E) for the wing porosity estimation. (**G**) Twelve frames of a free fall of an *A. campestre* seed, captured from the top, showing a complete autorotation. (**H**) Six frames of an *A. campestre* free fall laterally captured. (**I**) Frame captured from (H) for the estimation of the coning angle (β).

Natural *A. campestre* seeds were collected and analyzed as a basis of our approach in the biomimetic seed design. First, we define the main parts of the body of the natural seed. The natural *A. campestre* seed (Fig. 1A) is composed of the seed section (pericarp, labeled as “1” within Fig. 1A) and a single wing section (labeled as “2” within Fig. 1A).

All relevant morphometric characteristics of the natural *A. campestre* seeds are summarized in fig. S1. The average mass was 56 ± 11 mg (extracted from 10 seeds), while the center of mass (*C*_m_), estimated from seed sections (Fig. 1B) and analysis of the linear density, i.e., mass per unit length (*32*) (Fig. 1C), was in the range of 0.252 ± 0.042 of the seed lengths (*L*) (*32*). The average value of the wing surface (*S*), estimated from wing image binarization, was 173 ± 21 mm^2^ (Fig. 1D and fig. S2). Consequently, the wing loading [*W*/*S*; with *W* (weight) = 0.549 × 10^−^^3^ ± 0.1 × 10^−3^ N] was 3.17 ± 1.01 N/m^2^.

*A. campestre* single wings were also histologically analyzed to investigate the properties and distribution of the internal structure that facilitates its wind dispersal. Transversal sections of the single fibrous wing were analyzed using white light and fluorescence microscopy. The internal structure of the wing is characterized by extensive aerenchyma interrupted by vascular formations (fig. S3). Aerenchyma, which can be found in roots and shoots, is a plant tissue with large intracellular spaces (*33*). This tissue might allow gas exchanges under wetland conditions or, in the case of samara seeds, to facilitate their aerial dispersion.

The lignin distribution within the natural seeds was investigated via fluorescence microscopy using UV 355-nm excitation wavelength. The lignin is able to absorb this excitation source and emit within the blue wavelengths (*34*). The fluorescence images reveal that the vascular vessels are composed of lignified structures (fig. S3). Furthermore, cellulose distribution with Alcian blue dye was also observed in the transverse sections. Cellulose was mainly localized in the cells anchoring the vascular structure to the surrounding tissues (Fig. 1E and fig. S4, A to E). In addition, binarizing the images illustrated in Fig. 1E and fig. S4 (A to E), the porosity of the tissues of the wing could be extracted as reported in Materials and Methods (Fig. 1F and fig. S4, F to J). The porosity of the tissue is at high levels reaching values in the range of 70.5 ± 5.1% (for five samples).

The analysis of the aerodynamics of *A. campestre* seeds was specifically relevant for the measurements of key parameters for flight characterization, such as laboratory descent speed (*v*_d_), rotational velocity (Ω), wing tip speed (*v*_t_), and conic angle (β) (*12*, *32*). On the basis of this analysis, the average descent speed of the natural seeds was in the range of 1.04 ± 0.11 m/s. This value of the speed is consistent with the value reported for another type of natural maple seed, such as the *Acer platanoides* (Norway maple) seed with higher mass and length, reaching an average descent speed of 1.10 ± 0.24 m/s (*32*). The rotational velocity (Ω) was 160.5 ± 23.3 rad/s (roughly 25.5 ± 3.7 rotations/s) (movie S1). Twelve frames and one complete rotation during a free fall of the *A. campestre* seed are reported in Fig. 1G). The wing tip speed (*v*_t_) resulted in 3.94 ± 0.57 m/s, and it was calculated as (*32*)

$$v_{t}=\text{Ω}\;*\;R$$ (1)

where

$$R=L_{w}+\frac{1}{2}\;L_{p}$$ (2)

where *L*_w_ and *L*_p_ are the length of the wing and the length of the pericarp, respectively (fig. S1). The measured conic angle β was around 25.2° ± 3.2° (Fig. 1, H and I, and movie S2). This value matches the results reported for the rotary seeds (*10*) and ranges from an angle of 15° to 30°.

Reynolds numbers (*Re*) were calculated following Green (*35*):

$$Re=\frac{v_{d}\times w_{w}\times \rho_{\mathrm{air}}}{\nu_{\mathrm{air}}}$$ (3)

where *v*_d_ is the descent speed (1.04 ± 0.11 m/s), *w*_w_ is the width of the wing (0.0101 ± 0.0008 m), ρ_air_ is the density of the air (1.204 kg/m^3^), and ν_air_ is the kinematic viscosity of the air [1.81 × 10^−5^ kg/(m·s)].

Drag coefficients (*C*_D_) were calculated following Yoon *et al.* (*7*):

$$C_{D}=\frac{m\times g}{2\times \rho_{\mathrm{air}}\times {\left(v_{d}\right)}^{2}\times S}$$ (4)

where *m* is the mass (5.6 ± 1.1 × 10^−5^ kg), *v*_d_ is the descent speed (1.04 ± 0.11 m/s), *S* is the wing surface (1.73 ± 0.21 × 10^−4^ m^2^), *g* is the gravitational acceleration (9.81 m/s^2^), ρ_air_ is the density of the air (1.204 kg/m^3^).

The resulting *Re* and *C*_D_ values were 699 ± 129 and 4.87 ± 2.58, respectively. All morphometric and aerodynamic parameters of the natural seeds are summarized in fig. S1 and table S2 and were then used to design artificial seeds.

### Manufacture and characterizations of the artificial *A. campestre* seeds

A simplified scheme of the 3D printed process of the artificial seeds involves (fig. S5): (i) drawing of the contours from the natural seed, (ii) creation of a vector file, (iii) creation of a 3D CAD model, and (iv) printing PLA or its composites to obtain the I-SeedSam inspired by *A. campestre*. We chose PLA due to its biocompatibility, sufficient optical transmittance in the Vis, and its refractive index that matches that of the photoluminescent particles (*26*).

**Fig. 2.**
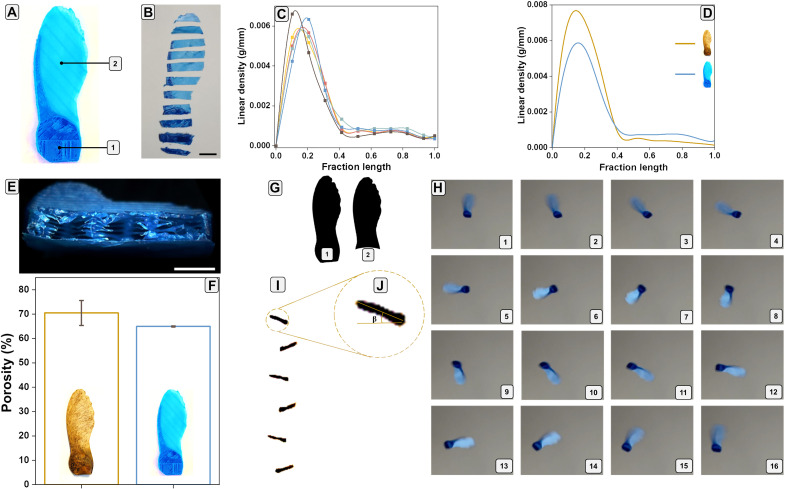
Artificial *A. campestre* seed morphometric and aerodynamic characterization. (**A**) Picture of an artificial *A. campestre* seed showing the nut or pericarp (as 1) and wing (as 2). (**B**) Seed sectioning. (**C**) Linear density (in grams per millimeter) of five artificial *A. campestre* seeds after sectioning. (**D**) Average linear density (in grams per millimeter) trends of natural and artificial *A. campestre* seeds. (**E**) Inner and porous structure of the printed artificial pericarp. Scale bar, 2 mm. (**F**) Porosity comparison between the natural acer seed wing and the artificial acer seed pericarp. (**G**) Image binarization of (A) with pericarp (1) and without pericarp (2) for the wing surface (*S*) and loading (*W*/*S*) estimation. (**H**) Sixteen frames of a free fall of an artificial *A. campestre* seed, captured from the bottom, showing a complete autorotation. (**I**) Six frames of an *A. campestre* free fall laterally captured. (**J**) Frame captured from (I) for the estimation of the coning angle (β) estimation.

The 3D printed artificial seed is illustrated in Fig. 2A. Infill of the pericarp (labeled as 1 within Fig. 2A) was set at 5%, while the infill of the wing (labeled as 2 within Fig. 2A) was set at 100%. For the wing, we set the 100% infill considering the achieved thickness of 0.05 mm, which is the lowest resolution limit of the applied 3D printer (Prusa i3 MK3S) (*36*). We varied the printing parameters to produce artificial seeds with similar sizes and weights as those of the natural flying seeds. Morphological details of the artificial seeds are reported in table S2, while pictures of the artificial pericarp and wing sections are reported in fig. S6.

The average mass of the artificial seeds was 55 ± 5 mg (number of samples = 8), while the center of mass (*C*_m_) estimated from seed sections (Fig. 2B) and analysis of the linear density (Fig. 2C) was around 0.19 ± 0.02 of the seed lengths (*L*) (for five samples). Both values were in the range of the characteristics of the natural seeds (table S2). The agreement between the *C*_m_ values is also confirmed by the trends of the average linear densities of the natural and artificial *A. campestre* seeds (Fig. 2D). The average porosity of the artificial pericarp (Fig. 2E) was estimated from weight measurements, resulting in a value in the range of 65.0 ± 0.2%, derived from the infill set at 5%. This value is comparable to the porosity estimated for the wing structure of the natural seed (70.5 ± 5.1%; Fig. 2F). Then, we estimated the average values of the wing surface (*S*) and the wing loadings (*W*/*S*) by wing images binarization. The wing surface (165 ± 2 mm^2^) (Fig. 2G and fig. S7) and the wing loading (3.3 ± 0.3 N/m^2^) agreed with the ones of the natural seeds (table S2).

From the aerodynamic point of view, the artificial seeds displayed an average descent speed (*v*_d_) of 1.04 ± 0.09 m/s, matching well with the value extracted for the natural seeds (1.04 ± 0.11 m/s). On the other hand, the rotational velocity Ω and the wing tip speed *v*_t_ were 107.1 ± 16.3 rad/s and 2.64 ± 0.41 m/s, respectively (Fig. 2H and movie S3). The measured coning angle β was 23.3° ± 6.3°, and the value was consistent with the ones reported for the natural *A. campestre* seed (within 20.5° ± 8.3° range) (Fig. 2, I and J, and movie S4).

Reynolds number (*Re*) was calculated considering *v*_d_ and *w*_w_ equal to 1.04 ± 0.09 m/s and 0.0095 ± 0.0004 m, respectively, and Eq. 3. The drag coefficient (*C*_D_) was calculated considering *v*_d_, *m*, and *S* equal to 1.04 ± 0.09 m/s, 5.5 ± 0.5 × 10^−5^ kg, 1.65 ± 0.02 × 10^−4^ m^2^, respectively, and Eq. 4. The resulting *Re* and *C*_D_ values were 654 ± 84 and 5.02 ± 1.39, respectively. Both *Re* and *C*_D_ values resulted statistically equal for natural and artificial seeds. All aerodynamic and morphometric parameters of the artificial and natural seeds are compared in table S2.

Figure S8 shows how morphometric and aerodynamic parameters of the artificial *A. campestre* seeds are affected by size variation, i.e., 0.5× and 2× compared to natural ones. The 2× type had a nearly doubled measured descend speed at 1.82 ± 0.33 m/s, while the 0.5× type was closer at 0.97 ± 0.09 m/s. According to these data, the bioinspired size seems to be a good compromise between a low descent speed (greater dispersion with the wind) and sufficient wing and pericarp surface for finding the flier (after perspective launches from drones) and reading the fluorescence from the pericarp.

**Fig. 3.**
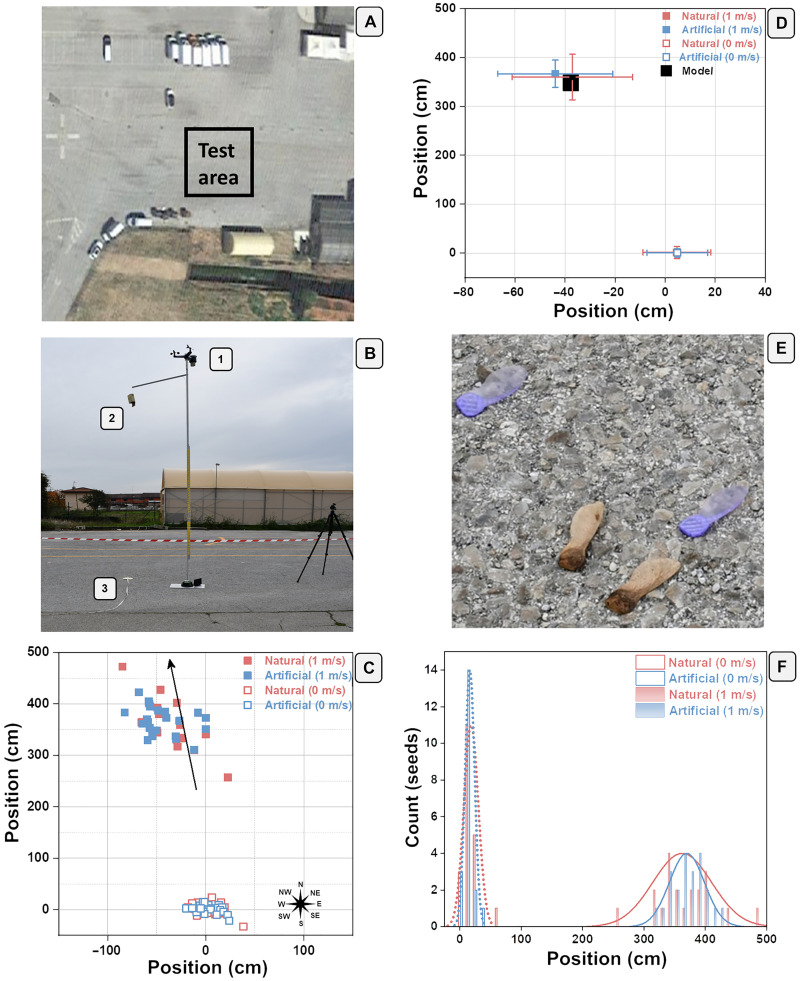
Natural and artificial *A. campestre* seed on-field release and dispersion tests. (**A**) Picture of the selected area (concrete parking lot) for the on-field test. Side of the square area is 20 m. (**B**) Setup for the on-field test release consisting of a weather station for the measure of the wind speed and direction placed at 3.8 m (1), a seed holder placed at 3.5 m from the ground (2), and a goniometer and meter for the seeds’ angle and position measurements (“3”). (**C**) Diagram showing the 20 natural and 20 artificial seed releases and positions with no wind condition (0 m/s) and under a wind speed of 1.0 ± 0.1 m/s coming from southeast. The black arrow shows the wind direction. (**D**) Diagram showing the average natural and artificial position originating from (C) with no wind condition (0 m/s) and under a wind speed of 1.0 ± 0.1 m/s. The square black symbol stands for the predicted distance of dispersal in agreement with the simple ballistic model (*38*). (**E**) Picture showing the release of natural and artificial *Acer* seeds. (**F**) Artificial and natural seed position distribution related to the experiments reported in (C) and (D).

For the abovementioned reasons, we run wind dispersal experiments outdoors (Fig. 3A) with the bioinspired artificial *A. campestre* seeds. We tested the dispersal performance of the natural and artificial seeds using a weather station (Fig. 3B) to measure the wind speed and direction (movie S5).

The wind velocity measured at the time of the release was 1 ± 0.1 m/s coming from southeast. The average distance of dispersion for the natural and artificial seeds was statistically identical, 362 ± 48 and 370 ± 29 cm, respectively, for 20 samples each (Fig. 3, C and D). In addition, the dispersion features of the natural and artificial seeds were tested under a wind velocity of 0 m/s. The seeds conducted the same dispersion features, achieving 18 ± 12– and 16 ± 8–cm average distance of dispersion, for 20 samples of natural and artificial seeds, respectively.

The main morphological and aerodynamic parameters that influence dispersion were the wing loading and the descent speed, which had roughly the same values for natural and artificial seeds (table S2). The obtained distances under a wind speed of 1.0 m/s were also in good agreement with the simple ballistic model used in dispersal studies of natural seeds (*37*, *38*)

$$x_{p}=H\frac{v_{w}}{v_{d}}$$ (5)

where *x*_p_ is the predicted distance, *H* is the height of release (3.5 m), *v*_w_ is the wind velocity at 3.5 m (1 m/s), and *v*_d_ is the descent speed of the seed (1.04 m/s), recorded indoors. We assumed the absence of turbulences as the wind speed remained constant for the duration of the fall (a few seconds), and we considered the release from the box as from a point source. We assumed the value of the dimensionless parameter θ, which characterizes the dispersion behavior of the seeds, to be 1, which is the value expected for heavy seeds (*38*). The predicted distance of dispersal was 337 ± 67 cm (Fig. 3E), which was a good estimation of the real mean distance for both natural and artificial seeds, within 362 ± 48– and 370 ± 29–cm ranges, respectively. The dispersal curves of the natural and artificial seeds are depicted in Fig. 3F. We note that under a wind speed of 1.0 m/s, the distribution is quite narrow, especially for the artificial seeds, as they had less variance in their morphology. Within 3 to 4 m, the natural and artificial seeds fell around 80 and 86%, respectively. These data allow the prediction with a certain degree of accuracy the distance and the distribution of the great majority of the released seeds.

Drones can be used to release the artificial *A. campestre* seeds in the environment (movie S6). A drone was equipped with a custom-designed seed container with wireless release control. Seeds release tests were carried out at two different heights [i.e., 3 and 5 m with no wind (0 m/s) conditions]. The average distance from the take off point was 48 ± 26 cm (number of samples = 5, one release) and 113 ± 36 cm, (number of samples = 14, three releases with five seeds, one seed was lost), respectively. The results agree with previously reported data with the weather/release static station (16 ± 8 cm) considering the drone oscillation/reposition and the propellers turbulence. The reported results demonstrate the suitability of the developed drone-based dynamic system for seed release.

### Preparation and characterizations of fluorescent composites

Our results indicate that the 3D printed, PLA-based artificial seeds can perform well as wind-deployable seeds. In the following, we incorporate a sensor material within the artificial seeds to enhance the passive bulk material with temperature sensing capabilities without degrading the aerodynamic properties and low environmental impact. To this end, we (i) prepared PLA-based fluorescent composites, (ii) created fluorescent filaments via melt processing that are suitable to the 3D printing approach introduced above, and (iii) produced fluorescent flying *A. campestre* seeds (I-SeedSam).

We filled the PLA with lanthanide-doped powders that act as upconverting emitters by absorbing low-energy NIR wavelengths and convert this energy into high emitting wavelengths (*29*). We selected Er^3+^ and Yb^3+^ in a hexagonal NaYF_4_ host with good crystallinity (fig. S9A) and low vibration modes (fig. S9B) to create strong fluorescence that can be detected in the field (*29*). The amount of the lanthanide ions within the host was 3 mol % of Er^3+^ and 20 mol % of Yb^3+^ to prompt a dominant green emission that is eye-safe and easy to detect using current state of the art technologies. When irradiated at 980 nm, these materials emit at around 520 and 540 nm (green wavelengths) and 660 nm (red wavelength) (fig. S10A). These wavelengths are compatible to contactless read out by drones equipped with fLiDAR (fluorescence light detection and ranging) (*39*). Sources for NIR excitation are low in cost due to their use in communication and safer to the human eye than UV or Vis excitation sources at comparable energies (*29*). In addition, NIR lights are less susceptible (around fourfold) to solar irradiation than UV light (*40*), used on current fliers based on organic molecules (*7*). The green emissions can be detected using readily available, sensitive red-green-blue charge-coupled device sensors.

The upconversion process involves two photons as determined by the relationship of the intensities of the emissions and the power of the laser applied (fig. S10B). Briefly, Yb^3+^ ions absorb the laser and can populate the energy levels of Er^3+^ ions via energy transfer mechanisms (*41*). Because of this, the Er^3+^ ion is excited to the higher ^4^F_7/2_ excited level, from which consecutive nonradiative processes can populate lower energy levels, such as ^2^H_11/2_, ^4^S_3/2_, and ^4^F_9/2_, each of them radiatively relaxing to the ground state ^4^I_15/2_ to generate emissions at 520, 540, and 660 nm, respectively (fig. S10C).

We varied the concentration of the lanthanide-doped materials within the PLA polymer to maximize the signal-to-noise ratio of the fluorescence. A solvent evaporation process (fig. S11) was used to efficiently prepare a systematic series of filling ratios in the polymer matrix (PLA). Dispersions of powder and PLA matrix at varying ratios were prepared in an apolar solvent. The dispersion was deposited into a template and formed solid, nonporous samples upon evaporation of the solvent. We obtained five round and opaque fluorescent composite samples with diameters of 1.75 cm and thicknesses of 0.25 cm (an example of a composite is illustrated in fig. S12) that were characterized by fluorescence spectrometry. A filling ratio of 10 wt % displayed the highest intensity of the green emissions of the lanthanide ions (fig. S13). The dominant emission was centered around 540 nm, and a weak red emission was centered around 660 nm, with the former wavelength being around eightfold higher compared to the latter wavelength (fig. S14). A typical picture of this composite under NIR irradiation displays a bright green emission (fig. S14).

### Production and characterizations of the fluorescent artificial seeds

We used melt processing to prepare fluorescent filaments with an optimal filling ratio as base material for temperature-sensing artificial seeds. The fluorescent materials and the polymeric matrix were mixed and introduced into an extruder operating at 453 K. The hexagonal phase of the NaYF_4_ host is thermally stable (*42*), and no structural degradation is expected. Thermogravimetric analysis revealed that the structure is stable until 1273 K, with a weight loss of only around 4%, starting after 873 K (fig. S15).

Five meters of 2-mm-diameter fluorescent filament were extruded using 40 g of fluorescent material and 400 g of PLA. We performed quality control using a 980-nm laser during different stages of filament extrusion. Movies were recorded at the nozzle (movie S7) and during the collection of the filament for further 3D processing (movie S8) that illustrates the bright green emission arising from the fluorescent materials. The emission was homogeneous and constant in intensity, confirming a uniform distribution of the fluorescent materials within the filament and no degradation.

**Fig. 4.**
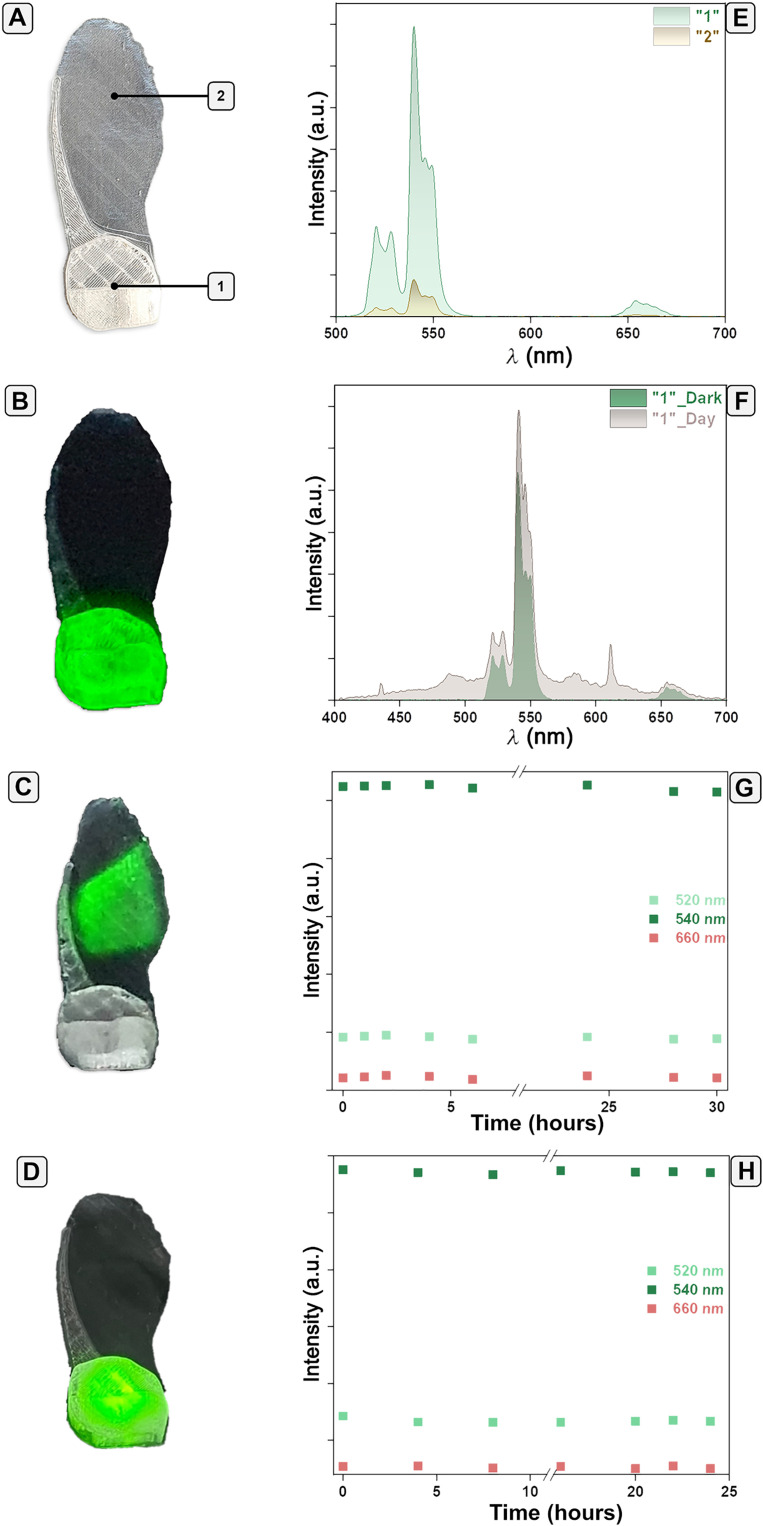
Thermometric characterization of the fluorescent I-SeedSam. Digital images of (**A**) a fluorescent artificial *A. campestre* seed under daylight, highlighting the pericarp (under 1) and the wing (under 2), (**B**) pericarp of the seed under NIR laser in the dark, (**C**) wing of the seed under NIR laser in the dark, and (**D**) pericarp of the seed under NIR laser in the daylight. Photoluminescence spectra of (**E**) the pericarp and wing of the seed recorded in the dark and (**F**) the pericarp recorded in the dark and daylight. Stability testing of the fluorescent seeds under (**G**) continuous NIR irradiation and (**H**) continuous sunlight irradiation. The photoluminescence spectra were recorded using the optical setup described in luminescent thermometry with fluorescent artificial flying seed section. a.u., arbitrary units.

The fluorescent filaments were then used to 3D print fluorescent artificial *A. campestre* seeds (I-SeedSam; Fig. 4A) via fused deposition modeling, as depicted in fig. S5. The I-SeedSam had the same morphometric characteristics, design, and mass (56.6 ± 1.1 mg, number of samples = 7) and the same descent speed [descent speed (*v*_d_) = 1.06 ± 0.02 m/s], as the nonfluorescent artificial *A. campestre* seeds (table S2). Because of this, we assume the same dispersion properties over the wind. Illumination with a 980-nm laser triggered the Vis photoluminescence of the lanthanide-doped composite. We compared the intensities of the emissions at the pericarp (Fig. 4B) and the wing (Fig. 4C) of the I-SeedSam by recording them with the same acquisition parameters (excitation source of 980 nm, power density around 0.4 W/cm^2^, and integration time of 1 s) in dark and daylight. The intensity of the emission at the pericarp was approximately 10-fold higher than that of the wing (Fig. 4E). The pericarp is approximately 1 mm in thickness, and the wing is only 0.05 mm, which strongly affects emission from the translucent sensor material. A comparison of the pericarp signal in dark and day light indicated that the characteristic green characteristics lines were still detectable, while the red emission was within the noise level (Fig. 4F).

We investigated the stability of the printed I-SeedSam by acquiring photoluminescence spectra while continuously irradiating with 980-nm laser light at a power density of 0.4 W/cm^2^. It is known that lanthanide-doped materials, composed of Yb^3+^ ions, can degrade via self-heating at high power densities (*43*). The intensities of the emissions of the Er^3+^ ions after irradiating for 30 hours (Fig. 4G) were within 99% of the original value. Exposure to a sun-light lamp tester at approximately 1 m (equivalent to 2500 lux) for 24 hours to simulate sunlight in the field indicated no degradation with time, as the intensities of the emissions were within 97% of the original values (Fig. 4H).

### Luminescent temperature sensing with the fluorescent artificial seeds

The fluorescent flying seeds were tested as luminescent thermometers, exploiting the temperature dependence of their photoluminescence (*23*). We extensively tested and validated the thermometric performance of the I-SeedSam to account for their sensitivity, reproducibility, and reliability as a function of different conditions. We used the FIR method to quantify the thermal response. This technique is not influenced by the optical setup, concentration of the nanoparticles, or external illumination, e.g., by sunlight (*23*). The emissions were monitored on a temperature-controlled stage within the range from 268 to 313 K (i.e., from −5.15° to 39.85°C) to mimic environmental temperatures. We calculated (i) the thermometric parameter (∆ or FIR), (ii) the relative thermal sensitivity (*S*_rel_), (iii) the temperature resolution (δ*T*), and (iv) repeatability (*R*) to account for the performance of the I-SeedSam as thermometers.

**Fig. 5.**
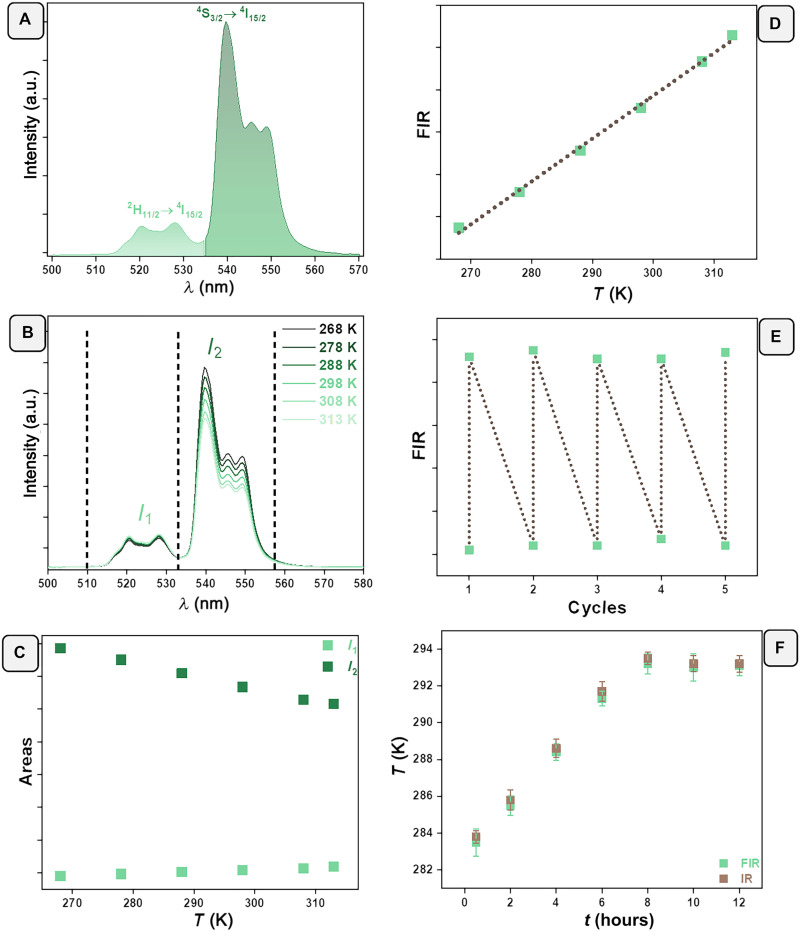
Thermometric performance of the fluorescent I-SeedSam. (**A**) Selected wavelength ranges for luminescent thermometry, (**B**) temperature dependence of the intensities of the selected wavelengths, (**C**) evolution of the integrated areas of the selected wavelengths as a function of temperature, (**D**) FIR as a function of temperature (the line is a fit to Eq. 4), (**E**) repeatability (*R*) of detected temperatures over five cooling/heating cycles, and (**F**) temperatures measured determined by I-SeedSam on topsoil (in green) and reference external infrared thermometer (in brown).

Luminescence thermometry of the I-SeedSam was based on the two green wavelengths at 520 nm (transition ^2^H_11/2_ → ^4^I_15/2_) and 540 nm (transition ^4^S_3/2_ → ^4^I_15/2_) (Fig. 5A). Their energy gap (∆*E*) relies within the range of the thermally coupled levels (*44*) from 200 to 2000 cm^−1^. Because of this, the FIR obeys a Boltzmann law (*45*)

$$\mathrm{FIR}=\frac{I_{1}}{I_{2}}=B\;*\;\exp\left(-\frac{\text{Δ}E}{k_{B}T}\right)$$ (6)

where *I*_1_ and *I*_2_ are the integrated area of the two green emissions at 520 and 540 nm (Fig. 5B), *B* the pre-exponential constant, ∆*E* the energy gap, *k*_B_ Boltzmann’s constant, and *T* the absolute temperature in kelvin.

Increasing temperature from 268 to 313 K barely affected emission around 520 nm, while the intensity around 540 nm decreased around 30% of its initial value (as illustrated Fig. 5, B and C, for the integrated areas). The ratio among these two integrated areas follows Eq. 4 (Fig. 5D). It is easy to extract the temperature using the parameters *B* and the experimental ∆*E* from (*45*)

$$\ln(\mathrm{FIR})=\ln(B)+\left(\frac{\text{Δ}E}{k_{B}T}\right)=\ln(B)+\left(-\frac{C}{T}\right)$$ (7)

with *B* and $C=\frac{\text{Δ}E}{k_{B}}$ fit from the experimental data. By plotting the logarithmic form of FIR as a function of the inverse of the temperature to the linear equation above, the values of the intercept and the slope could be extracted from the fitting (fig. S16A). The intercept and the slope are determined to be 0.92 ± 0.05 and 660 ± 15.7, respectively. Knowing these values, we can extract the value of the pre-exponential parameter *B* and the experimental energy gap ∆*E* (considering Eq. 5), which are 2.51 and 460 cm^−1^, respectively.

We evaluated the thermometric performance of the I-SeedSam in terms of *S*_rel_, δ*T*, and *R*. *S*_rel_ is a figure of merit that enables comparisons independent of the operating wavelengths, acquisition setups, and the nature of the materials (*23*, *45*). It indicates the maximum change of the thermometric parameter (FIR) per degree of temperature change as (*23*, *45*)

$$S_{\mathrm{rel}}=\frac{1}{\mathrm{FIR}}|\frac{\partial \mathrm{FIR}}{\partial T}|\times 100\%$$ (8)

After deducing the first derivative of the thermometric parameter as a function of temperature, the final expression of *S*_rel_ is as follows:

$$S_{\mathrm{rel}}=\;|\frac{\text{Δ}E}{k_{B}T^{2}}|\times 100\%$$ (9)

The maximal values of *S*_rel_ that we found were in the range of 0.92% K^−1^ at the lowest temperature (fig. S16B) in the same range as reported for other types of Er^3+^ green-emitting ions, regardless of the host or acquisition setups (*46*).

The temperature resolution δ*T* expresses the minimum change in temperature that a thermometer can detect (*23*, *45*)

$$\delta T=\frac{1}{S_{\mathrm{rel}}}\;\frac{\delta \text{Δ}}{\text{Δ}}$$ (10)

where $\frac{\delta \text{Δ}}{\text{Δ}}$ is the uncertainty of the determination of the thermometric parameter related to the detector applied (in this example, 0.5%) (*45*). The calculated temperature resolution was the range of 0.54 K for the lowest temperature and increased with temperature (fig. S16C).

The repeatability (*R*) indicates the ability of a thermometer to provide the same temperature measurements under the same identical conditions (*23*, *45*)

$$R=1-\frac{\max|{\text{Δ}}_{c}-{\text{Δ}}_{i}|}{{\text{Δ}}_{c}}$$ (11)

where ∆_c_ is the mean thermometric parameter and ∆_i_ is the value of each measurement of the thermometric parameter under the cooling/heating cycles. The value of *R* for five repeated measurements was around 98 ± 2% (Fig. 5E), indicating good repeatability.

Last, we emulated an environmental scenario in which the fluorescent artificial seeds fall on soil and measure its surface temperature (a fluorescent I-SeedSam on topsoil is illustrated in fig. S17). A fluorescent I-SeedSam was placed on top of a freshly collected soil sample within a custom-built optical setup. The soil was collected at low temperature in the morning (276 K, 2.85°C) that slowly increased in the laboratory that had a controlled room temperature of 294 K (20.85°C) and reached room temperature after 8 hours. The laser source was oriented perpendicular to the pericarp part of the fluorescent seed. Excitation with 980-nm laser induced photoluminescence that was recorded during the whole duration of the experiment (a total of 12 hours). The intensity ratios were then converted to temperatures using the calibration curve from FIR (Fig. 5D). The temperature matched that of the soil within 0.3 K (Fig. 5F) in a temperature range of 12 K.

## DISCUSSION

In summary, we demonstrated a strategy toward environmental physical sensing (e.g., temperature monitoring, as a proof of concept) using bioinspired flying seeds composed of fluorescent materials. We characterized and mimicked the properties of the natural *A. campestre* flying seeds and used 3D printing technologies to produce their artificial counterpart, with similar morphometric, aerodynamic, and wind dispersal characteristics. The artificial seeds were functional 3D printed in PLA: a biocompatible, compostable, recyclable, and slowly (bio)degrading material. The biodegradation/mineralization rate in soil is around 10% every 150 days according to an estimation reported elsewhere (*28*). This enables a sensing period of at least 4 years in the field, which is consistent with the envisioned use case scenarios. It is possible to use the processes described here or other related functional 3D printing methods and to mimic the design of the fluorescent I-SeedSams, using matrices that degrade faster, e.g. PLA/cellulose (*47*), cellulose acetate (*48*), and porous cellulose acetate (*49*).

Fluorescent lanthanide–doped materials, with temperature-dependent photoluminescence, were incorporated via melt processing before 3D printing. We created seeds with Er^3+^- and Yb^3+^-doped hexagonal NaYF_4_ as fluorescent materials that absorb NIR laser light and emit bright green emissions around 520 and 540 nm. The ratios of the emission intensities at the two wavelengths were ratiometrically analyzed to infer the environmental temperature. We successfully evaluated environmental scenarios such as continuous NIR or sunlight irradiation and topsoil temperature monitoring. In this way, we were able to advance the current state of the art using passive and biocompatible environmental sensors based on artificial fliers.

Although restricted to the development of fluorescent artificial *A. campestre* seeds, our single-step functional 3D printing approach is highly flexible. In perspective, other types of fliers (bioinspired or not) could be easily printed in mass production.

In the not too distant future, these fluorescent artificial seed–like fliers could be released by drones equipped with fLiDAR technology for remote and distributed monitoring of chemical-physical parameters (*50*). Although currently tested in thermal sensing, in the future, it is easy to imagine the incorporation of fluorescent particles sensitive to other environmental key parameters (e.g. humidity, CO_2_, or pollutants) (*22*) for a multiparametric wireless environmental analysis (*50*).

## MATERIALS AND METHODS

### From fluorescent lanthanide powders to fluorescent filaments

#### Chemicals

Lanthanide-doped microparticle powders were purchased from Sigma-Aldrich. The lanthanide ions (Er^3+^ and Yb^3+^) are doped within hexagonal NaYF_4_ hosts. The ratio of the dopants was 3 mol % of Er^3+^ and 20 mol % of Yb^3+^. The particle diameters ranged from 1 to 5 μm (d50), as determined by the supplier. PLA (natural and transparent, molecular weight of ~16,000 g/mol, melt temperature of 441 K, and density of 1.24 g/ml) polymer was purchased as pellets from BASF. The crystalline structure of the luminescent powders was characterized via x-ray powder diffraction using a D8 Advance diffractometer equipped with a copper source CuK_α_ radiation (λ = 1.54060 Å, 40 kV, 40 mA) operating within the 2θ range from 10° to 80°. The phonon energies of the microparticles were characterized by Raman spectroscopy using a Renishaw inVia microscope with a 532-nm unpolarized light within the wave number range of 100 to 2000 cm^−1^. Thermal gravimetric analysis of the luminescent powders was conducted in a Jupiter STA 449 F3 operated at a heating rate of 10 K/min under air and using Al_2_O_3_ as a reference material. The photoluminescence spectra were recorded using a FS5 spectrofluorometer from Edinburgh Instruments, equipped with an external NIR laser source operating at 980-nm wavelength. The emission spectra were acquired within the wavelength range from 400 to 800 nm with a resolution of 2 nm and an integration time of 0.5 s.

#### Preparation of fluorescent composites

The fluorescent composites were composed of the PLA, and the fluorescent materials were produced by solvent processing method. The concentration of the fluorescent materials within the polymer matrix was varied with the goal of optimizing the optical properties. Briefly, PLA (1 g) as pellets was dissolved in chloroform (around 10 ml) aided by vortexing and ultrasonication. Lanthanide-doped powders were added at different concentrations to the organic PLA solution. After continuous stirring, homogeneous dispersions were obtained. Then, around 2 ml of each composition was deposited into a Teflon template. The solutions were allowed to dry at room temperature to yield fluorescent sensor composites. The photoluminescence spectra of the composites were recorded using the same parameters described in the “From fluorescent lanthanide powders to fluorescent filaments” section for the powders. Photographs (fig. S14) of the fluorescent composites were acquired with a Nikon D3400 digital camera operating in manual mode with a 5.6 aperture, an exposure time of 1/20 s, and a sensor sensitivity setting of ISO 100.

#### Preparation of fluorescent filaments

Filaments were produced via melt processing by mechanically mixing PLA powder with 10 wt % of lanthanide microparticles in a speed mixer (Hauschild SpeedMixer DAC 150.3 SP) operating under 1000 rpm for 60 s. Per each batch, 40 g of fluorescent materials and 400 g of PLA were mixed. The mixture was introduced into a fully segmented and corotating twin-screw extruder (Process 11 Parallel from Thermo Fisher Scientific), equipped with a 2-mm-diameter nozzle. Fluorescent filaments were extruded at a screw speed of 159 rpm and a torque force of 94 Nm, with all heating zones operating at 453 K. Movies (movies S7 and S8) during the production process of the fluorescent filaments were recorded using an iPhone 14 Pro smartphone.

### From natural flying seeds to 3D printed fluorescent flying seeds

#### Morphometric and histological characterizations of the natural A. campestre seeds

*A. campestre* seeds were collected from an *Acer* plant in the rural area of Florence (Italy). Morphometric analysis of the seeds was carried out using a digital caliper (RS PRO 150 mm Digital Caliper 0.0005 in, 0.01 mm, Metric & Imperial, United Kingdom) with a resolution of ±0.01 mm and a digital microscope (KH-8700, Hirox, Japan). The mass of the seeds was measured out with an analytical balance (KERN ABS-N, Germany) with a resolution of ±0.0001 g. The experimental data were collected from a pool of 10 independent seeds.

The key morphological parameters for the flight performance of the seeds are (i) center of mass (*C*_m_), (ii) wing surface (*S*), and (iii) wing loading (*W*/*S*) (*32*). For the determination of the center of mass (*C*_m_), seeds were cut every 3 mm alongside their longitudinal axis with a lancet, and the resulting sections were weighted with an analytical balance (KERN ABS-N, Germany). The experimental data were collected from a pool of eight independent seeds. The mean curve of the linear density as function of the length was computed. The wing surface (*S*) was estimated from pictures of *A. campestre* seeds (10 seeds) captured with a camera (1280 × 800 pixels) of a Samsung A40 (South Korea) smartphone. Using ImageJ, the image was binarized, and the wing surface *S* was evaluated by counting the black pixels within a scale bar of 2 cm. The wing loading (*W*/*S*) was estimated considering the weight value (*W*) of the seeds and the wing surface (*S*).

The histological analysis of the wings of the *A. campestre* seeds was carried out by embedding the 5-mm cuts of the wings in paraffin and cutting them into 10-μm sections with a microtome (Leica microtome SM2010R), as described elsewhere (*51*). Alcian blue stain was used to analyze the cellulose localization within the transverse sections, while lignin distribution was detected by its autofluorescence in the UV (355 nm) range and by monitoring the emissions in the blue. The sections were observed with a white light epifluorescence microscope (Nikon Eclipse Ni-U, Japan), and the images were captured with the software NIS-Elements.

By analyzing different areas of the wing with scanning electron microscopy, we were able to obtain detailed images of the level of the porosity of the tissues. An estimation of the wing porosity (*P*_w_) in the thicker section was carried out by binarizing a scanning electron microscopy image with ImageJ software (*52*). The porosity of the wing (*P*_w_) was estimated by counting the black (*N*_black pix_) and white (*N*_white pix_) pixels, representing the voids and the material, respectively.

$$P_{w}=\frac{N_{\mathrm{black}\;\mathrm{pix}}}{N_{\mathrm{black}\;\mathrm{pix}}+N_{\mathrm{white}\;\mathrm{pix}}}$$ (12)

#### Determination of the aerodynamic performance of the natural A. campestre seeds in laboratory

The analysis of aerodynamics of natural seeds under laboratory conditions consisted of the measurements of key parameters that characterize the flight, such as (i) descent speed (*v*_d_), (ii) rotational velocity (Ω), (iii) wing tip speed (*v*_t_), and (iv) conic angle (β) (*12*, *32*). To determine the descent speed (*v*_d_), the *A. campestre* seeds were released from rest in a still air setting from a height of 2.95 m and allowed to fall freely. Tests were conducted in a laboratory without active ventilation. The flight of the seed was recorded by a camera of a Xiaomi Redmi Note 5 (China) with a resolution of 1920 × 1080 pixels. The mean *v*_d_ was calculated considering the time elapsed between the frame of the release and the frame in which the seed touches the ground. Each individual seed was tested three times (10 *A. campestre* seeds, each dropped three times, giving 30 drops in total).

The protocol described above was also applied for the measurement of the rotational velocity (Ω) and wing tip speed (*v*_t_). In this case, a camera of an Apple iPhone 12 Pro Max (USA) was used to record movies of a free fall at 240 frames/s (4.17 ms between frames) with a resolution of 1280 × 800 pixels. The angle variations (∆°) in the last second of free fall were measured by the frames with a digital goniometer software (ImageJ). The same protocol was also applied for the estimation of the coning angle β that depends on the balancing of the centrifugal force, acting on the distributed mass of the seed, the distributed weight of the seed, and the aerodynamic force that causes the driving moment around flapping *x* axis (*12*).

#### On-field release and dispersion as a function of wind (meteorologic station)

The on-field aerodynamic performance of natural *A. campestre* seeds was investigated. This test was conducted on a paved square located in Mercato Fiori e Piante della Toscana, (MEFIT), via Salvo D’Acquisto, 10/12, 51017 Pescia PT. The natural seeds (20 samples) were placed in a box that was lifted on a pole to a height of 3.5 m. The box was built of a magnetic door on the bottom side and tied with a magnetic rope that was pulled to release the seeds. Natural seeds were released simultaneously to be affected by the same wind conditions. A wind station Eurochron EFWS 2900 with a resolution of 0.1 m/s was used to measure the wind speed and its direction. The distance of each seed from the pole on the *XY* plane was measured with 20 m and the formed angle with a goniometer. The data were used to build a dispersal curve and a map of the seeds. The data were fitted to a simple ballistic model, and the fitting parameters were assessed.

#### 3D printing process of the artificial A. campestre seeds

For the design of the artificial samara seed, a top view picture of a natural *A. campestre* seed was coupled with the size analysis performed in the “Morphometric and histological characterizations of the natural *A. campestre* seeds” section. A vector file of the contours was extracted from the picture with Affinity Designer (trial version) and imported in the 3D CAD modeling software Siemens NX, where the design of the artificial samara was developed. For the fabrication, a fused deposition modeling process was used. The CAD model was converted in STL format and sliced with PrusaSlicer. The density of the internal infill chosen for the bulky part of the samara seed was 5%, and the printing speed was set at 20 mm/s. The 3D printer used was a Prusa i3 MKS (Prusa, Czech Republic), and the filament material for the printing was Prusament PLA Blend Royal Blue or Galaxy Purple (Prusa, Czech Republic). A nozzle with a diameter of 0.25 mm was used to obtain fine details and a wing thickness of 0.05 mm. The real porosity of the 3D printed seed pericarp (infill 5%) was evaluated by printing a version of the seed with a 100% infill and comparing the masses. The design, fabrication, morphological, and aerodynamic characterization of the artificial samara seeds was an iterative optimization process. It continued until the morphological and aerodynamic parameters obtained were comparable to the natural seeds. 3D printed artificial seeds with twice (2×) and half (0.5×) the naturally occurring size were printed using the same design and material and characterized as described in the previous paragraph “Determination of the aerodynamic performance of the natural *A. campestre* seeds in laboratory”. The fluorescent artificial *Acer Campestre* seeds were produced using the same 3D printing process but using the fluorescent filaments made of PLA described in the “Preparation of fluorescent filaments” section.

#### Morphometric and aerodynamic characterizations of the artificial A. campestre seeds

Morphometric and aerodynamic characterizations of the artificial *A. campestre* seeds are conducted within the laboratories and on-field using the meteorologic station (as described in the previous sections). On-field release experiments with artificial *A. campestre* seeds were also carried out in a private olive grove in Pietrabuona, Pescia (Italy). The drone (DJI mini, DJI, China, 249 g of mass) was equipped with a wireless and homemade release system in Teflon (movie S6). The tests were carried out at 3 and 5 m in height with no wind conditions (0 m/s). The distance of each seed from the take off point on the *XY* plane was measured with a yardstick. The data were used to build a map of the distribution of the seeds.

### Luminescent temperature sensing with fluorescent artificial flying seeds

The performance of the artificial flying seeds as luminescent thermometers was evaluated by monitoring the photoluminescence of the lanthanide-doped materials as a function of temperature. The artificial flying seeds were exposed to temperatures from 268 to 313 K on a cooling/heating stage (Linkam Scientific Instruments, THMS 600). The photoluminescence spectra were recorded using a FS5 Edinburgh Instruments spectrofluorometer, equipped with an external NIR laser source. The seeds were lying flat on the stage, with the NIR laser irradiating the pericarp of the seeds with a power density of approximately 0.4 W/cm^2^. Their photoluminescence was monitored within the wavelength range from 400 to 800 nm, using a resolution of 2 nm and an integration time of 0.5 s while heating or cooling at a rate of 1 K/s. The repeatability of the temperature-dependent response was tested by submitting the seeds to cooling and heating cycles (five in total) and recording the photoluminescence spectra during each cycle.

The readout of the fluorescent seeds from a distance was evaluated in a custom-built optical setup that emulates the situation in the field. The beam from an NIR laser operating at 980-nm wavelength (RLP-980-300 from Roithner Lasertechnik GmbH) was focused on the fluorescent seeds at normal incidence using a collimating lens (74-VIS from Ocean Optics) to reach a power density of around 0.4 W/cm^2^. The emitted fluorescence was collected using a 400-μm-diameter fiber (QP400-2-SRIBX from Ocean Optics) coupled with a high-resolution spectrometer (HB2000+ from Ocean Optics). The excitation light was removed using a 750-nm shortpass dichroic filter (FESHO750 from Thorlabs).

The fluorescent seeds were placed flat on a stage. The excitation source was aligned perpendicular to the seeds at approximately 100 cm. The fluorescence signal was collected by the fiber at an angle of 60° and a distance of 20 cm and entered the spectrometer, where it was continuously recorded at an integration time of 1 s. The stability of the fluorescence emission was evaluated by continuously irradiating the seeds with the NIR laser for 30 hours and measuring the emissions at constant temperature. Environmental conditions were mimicked by exposing the seeds to the light of a sunlight testing lamp at 10,000 lux (TL 50 from Beurer GmbH) at a distance of 100 cm. Photoluminescence was recorded as a function of time during 24 hours. The effect of soil was evaluated by placing the seeds on flat topsoil and freshly collected and without vegetation. The intensity of the fluorescence signal was monitored as a function of the temperature of the soil during a day within a laboratory room under air conditioner, without applying any external heating or cooling. The temperature of the soil was separately monitored using an infrared surface thermometer (IR 900 30-S from Voltcraft, Germany), pointing toward the position of the seeds on the topsoil, at a resolution of 0.1 K. Photographs of the fluorescent seeds were acquired with a Nikon D3400 digital camera, as described for the fluorescent composites.

## Supplementary Materials

### This PDF file includes:

Figs. S1 to S17 Tables S1 and S2 Legends for movies S1 to S8 References
