# Supplementary material for: A printed luminescent flier inspired by plant seeds for eco-friendly physical sensing
Source: Sci Adv. 2023 Nov 15;9(46):eadi8492. doi: 10.1126/sciadv.adi8492 (PMC10651124; doi:10.1126/sciadv.adi8492)
Supplement: Supplementary file 1 — Figs. S1 to S17 Tables S1 and S2 Legends for movies S1 to S8 References [file sciadv.adi8492_sm.pdf]

Science Advances
AAAS

Supplementary Materials for

A printed luminescent flier inspired by plant seeds for eco-friendly physical sensing

Kliton Cikalleshi *et al.*

Corresponding author: Stefano Mariani, stefano.mariani@iit.it; Tobias Kraus, tobias.kraus@leibniz-inm.de; Barbara Mazzolai, barbara.mazzolai@iit.it

Sci. Adv. **9**, eadi8492 (2023)
DOI: 10.1126/sciadv.adi8492

The PDF file includes:

Figs. S1 to S17
Tables S1 and S2
Legends for movies S1 to S8
References

Other Supplementary Material for this manuscript includes the following:

Movies S1 to S8

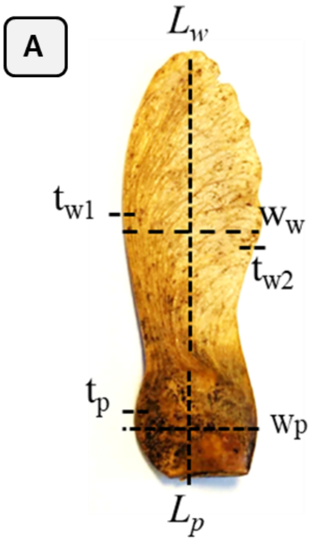

B

	Acer Seed
**Number of samples**	10
**Mass (mg)**	56 ± 11
**L_w (mm)**	20.3 ± 0.4
**w_w (mm)**	10.1 ± 0.8
**t_{w1} (µm)**	435 ± 33
**t_{w2} (µm)**	82 ± 12
**t_p (mm)**	3.5 ± 0.2
**L_p (mm)**	8.6 ± 0.9
**w_p (mm)**	8.6 ± 0.5

Fig. S1. Morphometry of the *Acer campestre* seeds. (a) Picture of an *Acer campestre* seed where: L_w is the length of the wing; w_w is the maximum width of the wing; t_{w1} and t_{w2} are the thicker and less thick thickness in correspondence of w_w; L_p and w_p are the length and the width of an *Acer campestre* pericarp; t_p is the thickness of the pericarp. The mass of the seed is also reported. (b) Morphometric analysis of the *Acer campestre* seed. Values are means ± s.d., with measurements from 10 samples.

Table S1. State of the art of the seed-inspired fliers for environmental sensing. The table summarizes the seed source of bioinspiration, the materials size and fabrication, the sensor integration strategy, the descent speed, the monitored environmental parameters, and the biocompatibility and/or biodegradability.

**Seed-inspiration**	**Material Size/Mass**	**Production and sensor integration**	**Aerodynamic performance**	**Monitored parameters and mechanism**	**Environmental impact**	**Ref**
Samara (genus *Acer*)	•Printed circuit board (PCB) in polyamide and metals<br>•Length: 12 cm<br>•Mass: 15 g	•PCB shaped in winged geometry<br>•Conventional electronic integration	Free fall descent speed:<br>0.65 m/s, 110 rpm	•Temperature, humidity, pressure<br>•Electronic wireless communication with antenna	Not biodegradable	*(16)*<br>*(53)*
Dandelion (*genus Taraxacum)*	•Parachute in polyimide, and metals for electronics<br>•Diameter: 28 mm, 55% fill<br>•Mass: 30 mg electronic components + 6.6 mg drag structure	•Laser cutting the thin polyimide films<br>•Ex-post integration of electronic components	Free fall descent speed 0.99 m/s	•Temperature, humidity, light, pressure, magnetic fields and acceleration<br>•Wireless electronic with antenna, solar cells, capacitor and RF switch.	Not biodegradable	*(5)*
Tristellateia australasiae (woody vines)	•PLGA and Cellulose<br>•Thickness of ~60 µm.<br>•Diameter about 4.2 mm; mass 1.5 mg	•Laser ablation of 2D precursors in PLGA<br>•Transfer onto a PDMS substrate and bonding of cellulose.<br>•3D shaping via heating<br>•Dyes integration via vacuum filtration method on the cellulose layer	Free fall descent speed<br>~ 0.7-1 m/s	•Humidity, pH, light exposure, and heavy metals<br>• RGB analysis of digital images	Biodegradable.<br>• Fungal biodegradation (*Postia placenta* and *Phanerochaete chrysosporium)* of the constituent materials of 3D colorimetric fliers in 12 weeks<br>• Cellulose mass loss was roughly the 2% while PLGA loss was roughly the 10% (*Postia placenta)*	*(7)*
Alsomitra Macrocarpa	•Potato paper, cellulose nanofiber-gelatin composite film (CNF:G) and shellac passive layer.<br>• Mass: 1.25 and 1.75 g, maximum width 140 mm	Plotter cutter + pressing into a 3D shape	Gliding rate of the natural seed 0.3-0.7 m/s	•PH sensing<br>•Colorimetric indicator protected by a hygroscopic actuator.	Biodegradable.<br>The tests were performed for 77 days at constant 58°C (50% weight loss after 77 days)	*(8)*
Samara (*Acer campestre*)	• PLA/ erbium (Er^{3+}) and ytterbium (Yb^{3+}) in a hexagonal $NaYF_4$ host (extruded filament)<br>• Weight 55±5 mg<br>• Length ~ 3 cm	Functional 3D printing of the I-SeedSam with the luminescent filament sensitive to the temperature	Free fall descent speed<br>~1.04 m/s	•Temperature sensing<br>•Wireless luminescent reading	Biocompatible, biocompostable and (bio)degradable.<br>Estimated biodegradation rate: roughly 4 years, considering a PLA mineralization rate in soil ~ 10% every 150 days as previously reported by *(28)*.	**This work**

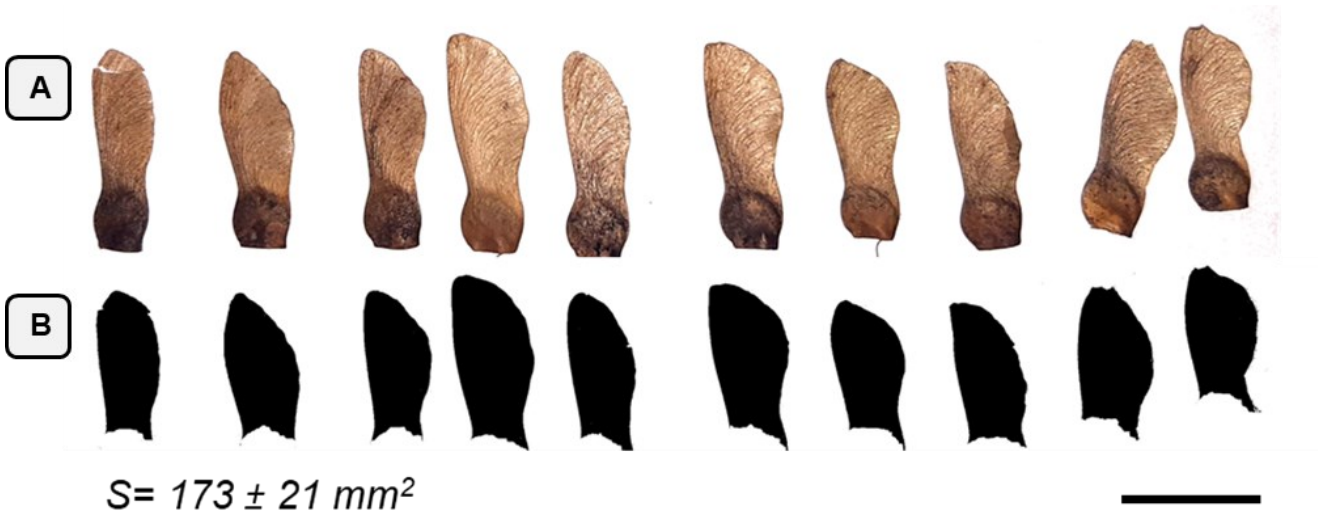

Fig. S2. *Acer campestre* seed wing surface. (a) Picture of ten *Acer campestre* seeds. (b) Image binarization of the wings reported in picture (a) for the evaluation of the wing surface and of the wing loading (N/m^2). The digital processing was performed with ImageJ. Scalebar is 2 cm.

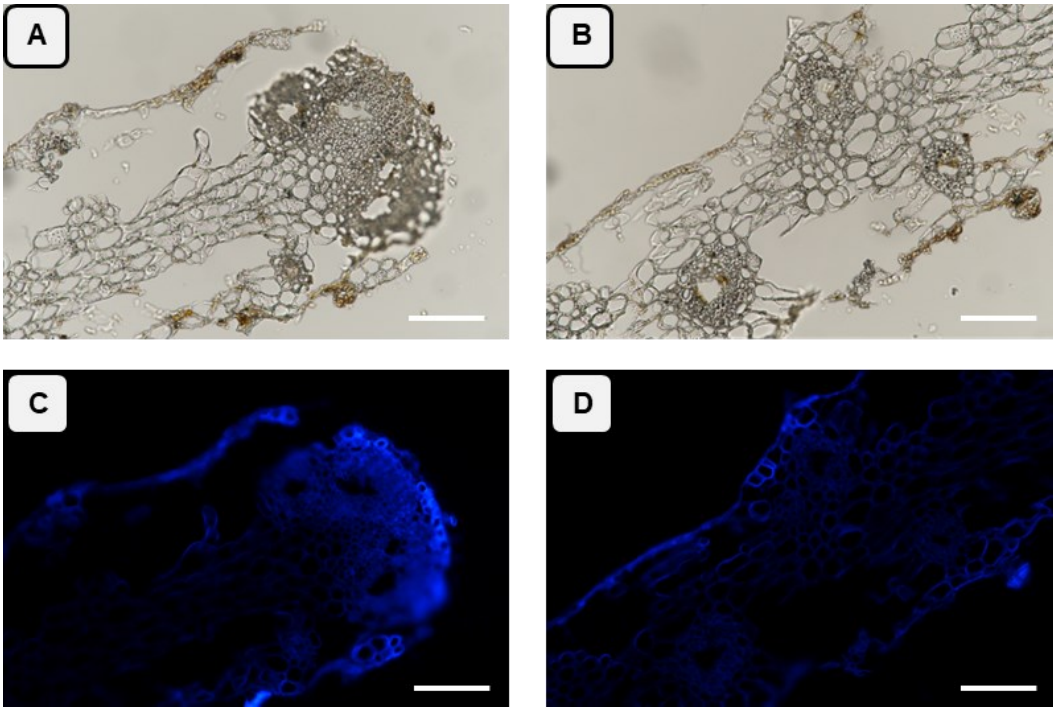

Fig. S3. Microscopy analysis of the *Acer campestre* seed wing. (a), (b) White image of a transversal section of the fibrous wing. (c), (d) Fluorescent image of (a) and (b) with lignin autofluorescence, excitation at 355 nm. Scalebar is 10 µm.

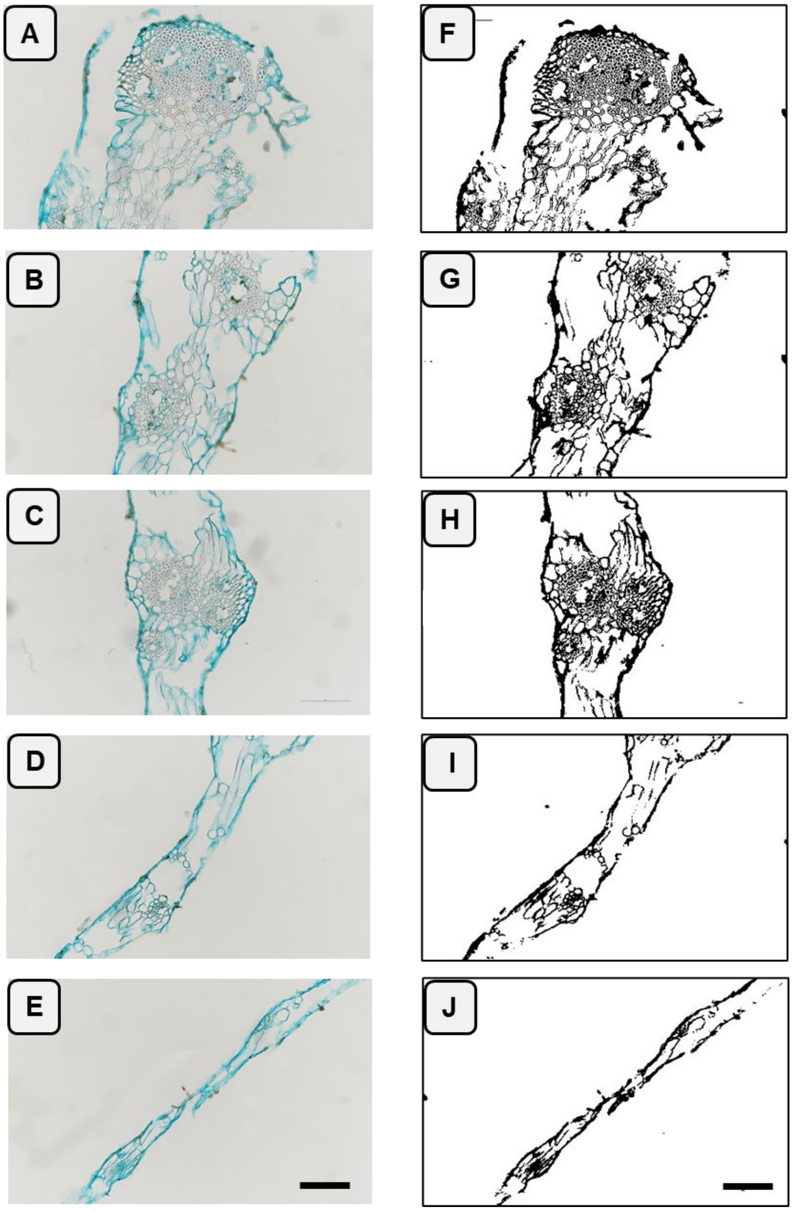

Fig. S4. Porosity of the *Acer campestre* wing. (a)-(e) Transversal section of the wing in different points stained with Alcian blue. (f)-(j) Binarized images from (a)-(e) for the wing porosity estimation.

Table S2. Morphometric and aerodynamic parameter of the natural and artificial *Acer campestre* seeds.

	**Natural *Acer campestre* seed**	**Artificial *Acer campestre* seed**
**N samples**	10	8
**Mass (*M*, mg)**	56 ± 11	55 ± 5
**Center of mass (C_m)**	0.25 ± 0.04 L	0.19 ± 0.02 L
**Wing surface (S, mm^2)**	173 ± 21	165 ± 2
**Wing loading (W/S, N/m^2)**	3.17 ± 1.01	3.3 ± 0.3
**Descent speed (v_d, m/s)**	1.04 ± 0.11	1.04 ± 0.09
**Rotational velocity (Ω, rad/s)**	160.5 ± 23.3	107.1 ± 16.7
**Wing tip speed (v_t, m/s)**	3.94 ± 0.57	2.64 ± 0.41
**Coning angle (β, °)**	20.5 ± 8.3	23.3 ± 6.3
**Re**	699 ±129	654 ± 84
**C_D**	4.87 ± 2.58	5.02 ± 1.39

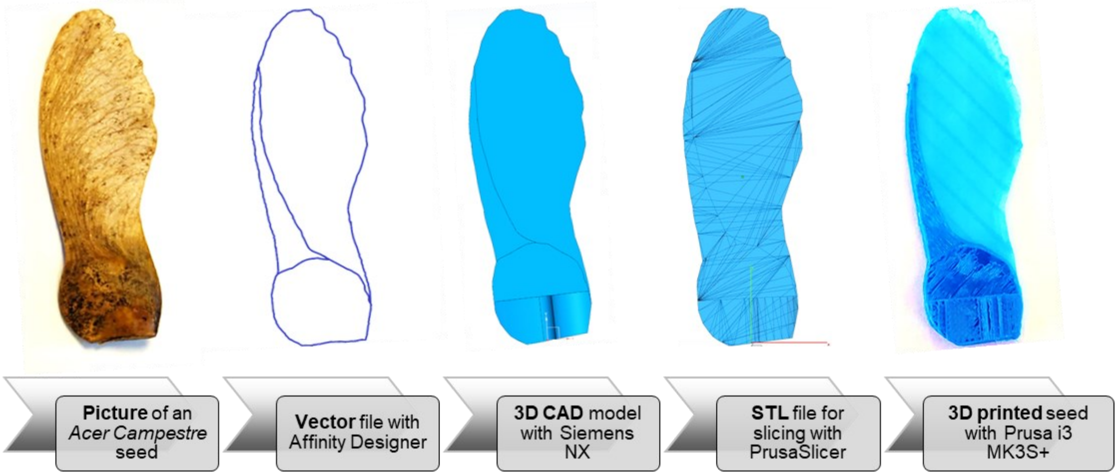

Fig. S5. Flow chart for the 3D printing of the artificial *Acer campestre* seed. It consist of: (i) *Acer campestre* seed; (ii) drawing of the contours; (iii) creation of a vector file; (iv) creation of a 3D CAD model, and (v) 3D printing of the artificial seed.

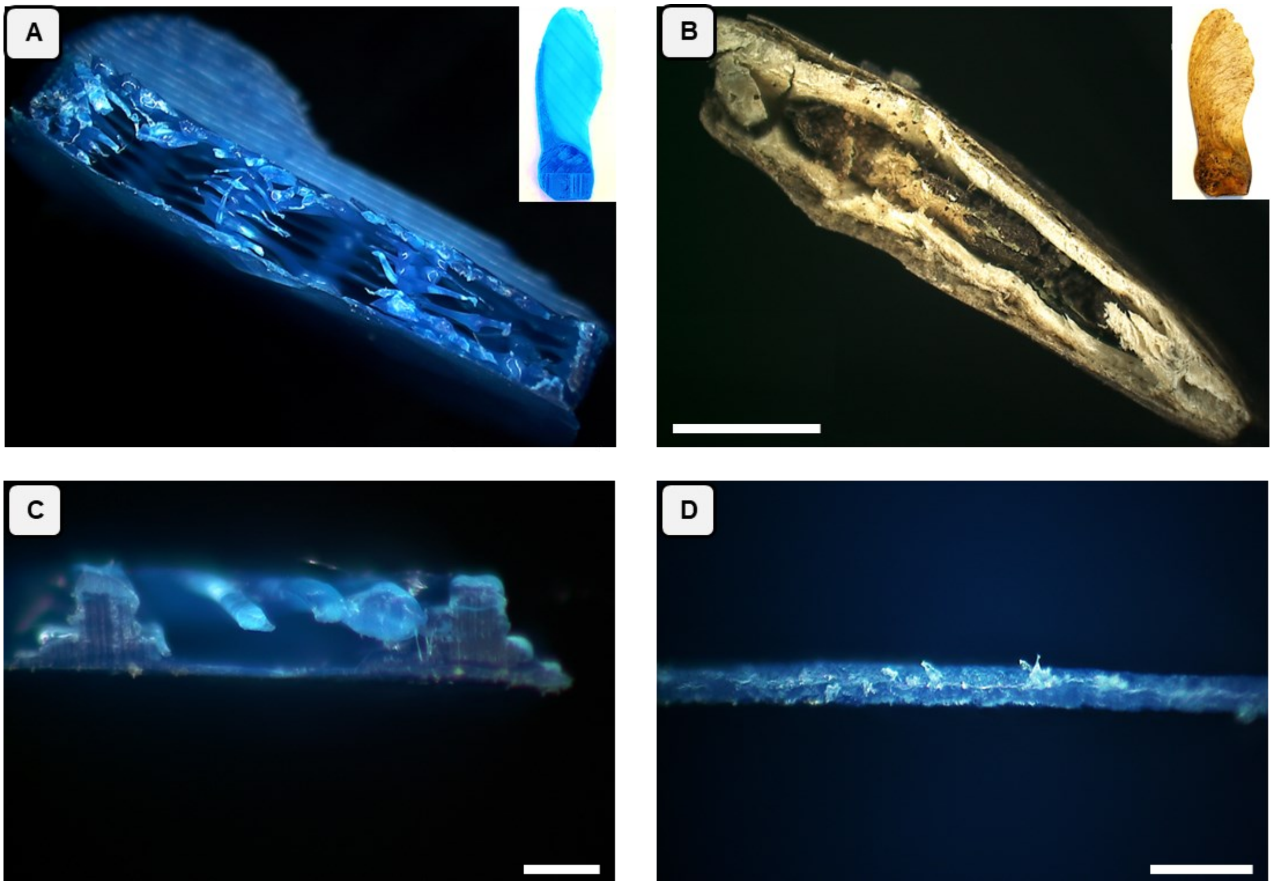

Fig. S6. Microscopy analysis of the artificial and natural *Acer campestre* wings and pericarps. (a) Section of the artificial and porous pericarp. (b) Section of the natural and porous pericarp. Scalebars for (a) and (b) are 2 mm. (c) Section of the artificial wing in the thicker section. Scalebar is 250 µm. (d) Section of the wing in the thinner section. Scalebar is 100 µm.

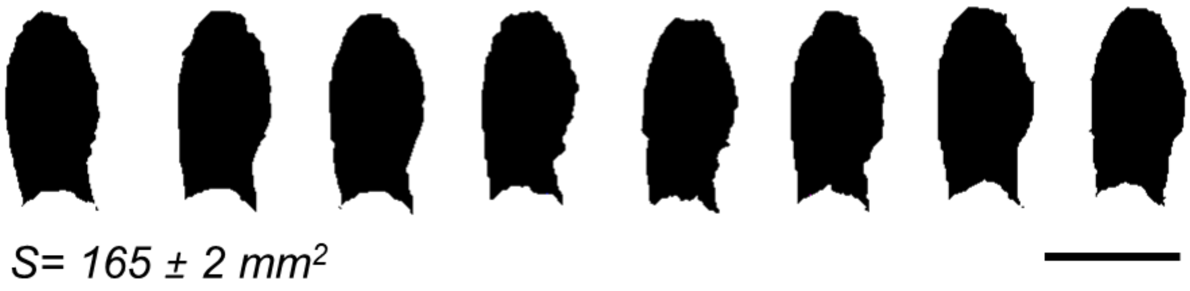

Fig. S7. Artificial *Acer campestre* wing surface. Image binarization of the artificial *Acer campestre* seed wings for the evaluation of the wing surface and of the wing loading (N/m^2). The digital processing was performed with ImageJ. Scalebar is 2 cm.

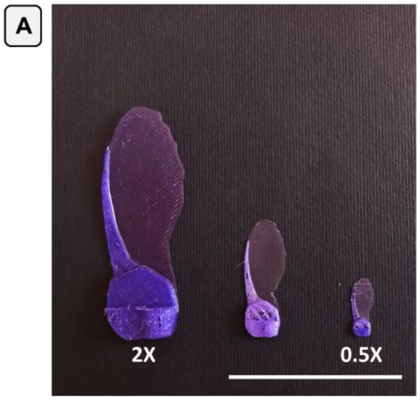

B

	2X		0.5X
Mass (M, mg)	599 ± 5	55 ± 5	11.2 ± 0.4
Wing surface (S, mm^2)	666 ± 20	165 ± 2	48 ± 3
Wing loading (W/S, N/m^2)	8.8 ± 0.3	3.3 ± 0.3	2.3 ± 0.2
Descent speed (v_d, m/s)	1.82 ± 0.33	1.04 ± 0.09	0.97 ± 0.09
Rotational velocity (Ω, rad/s)	68.3 ± 6.3	107.1 ± 16.7	161.1 ± 36.9
Wing tip speed (v_t, m/s)	3.50 ± 0.39	2.64 ± 0.41	2.04 ± 0.53

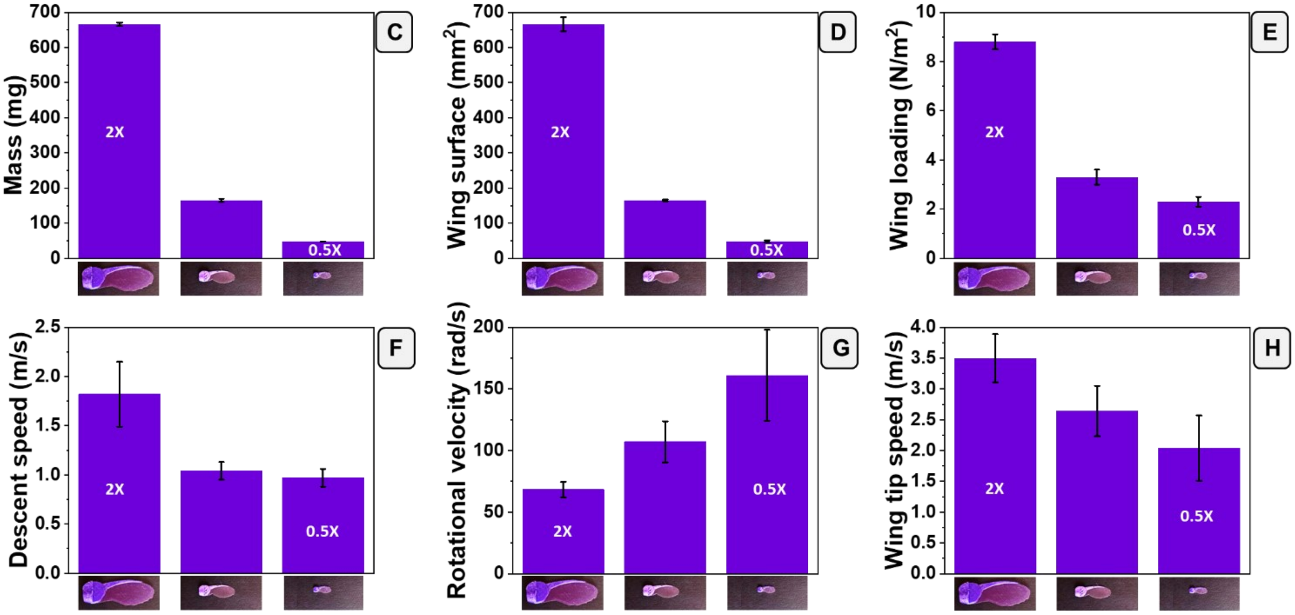

Fig. S8. Scalability of the artificial *Acer campestre* seed. (a) Picture of artificial Acer campestre seeds with different sizes: 2X and 0.5X the size of the seed in Fig. 2. Scalebar is 5 cm. (b) Morphometric and aerodynamic parameters of the artificial *Acer campestre* seeds with different sizes. (c)-(h) Comparison of the morphometric and aerodynamic performance for the artificial *Acer campestre* seed with different sizes: (c) mass; (d) wing surface; (e) wing loading; (f) descent speed; (g) rotational velocity; (h) wing tip speed (N samples: 8-19).

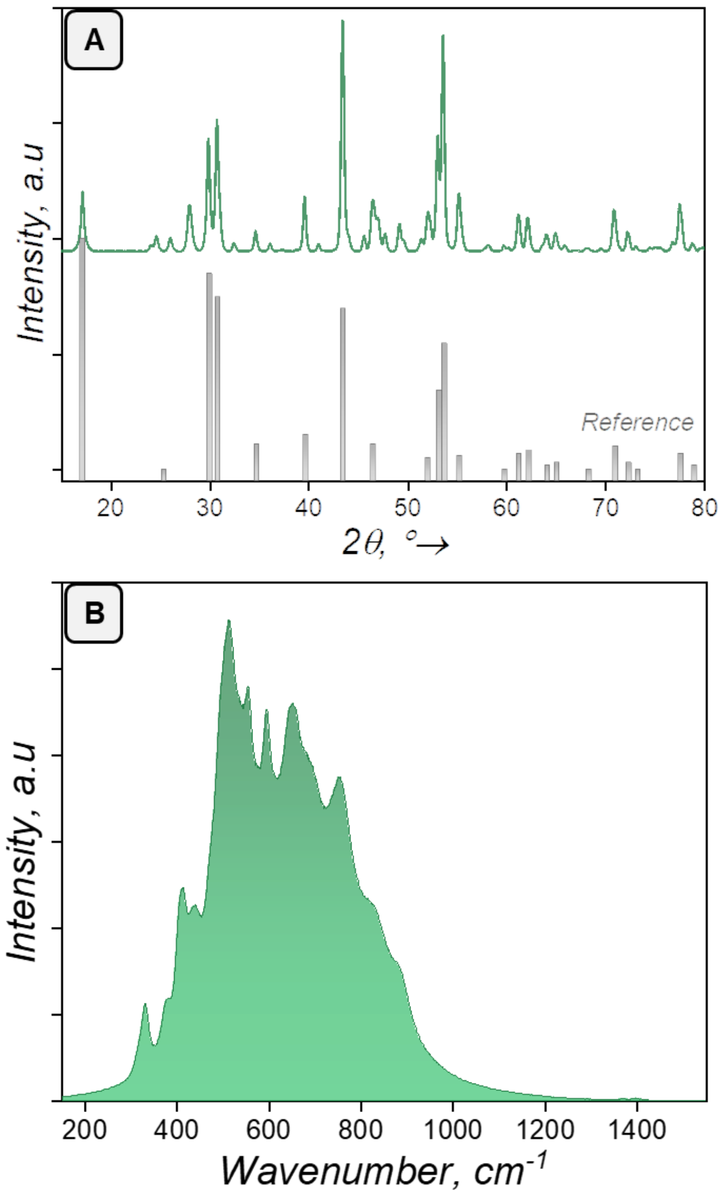

Fig. S9. Structural characterizations of $NaYF_4$ host. (a) X-ray powder diffraction, and (b) Raman vibration modes of $NaYF_4$ host.

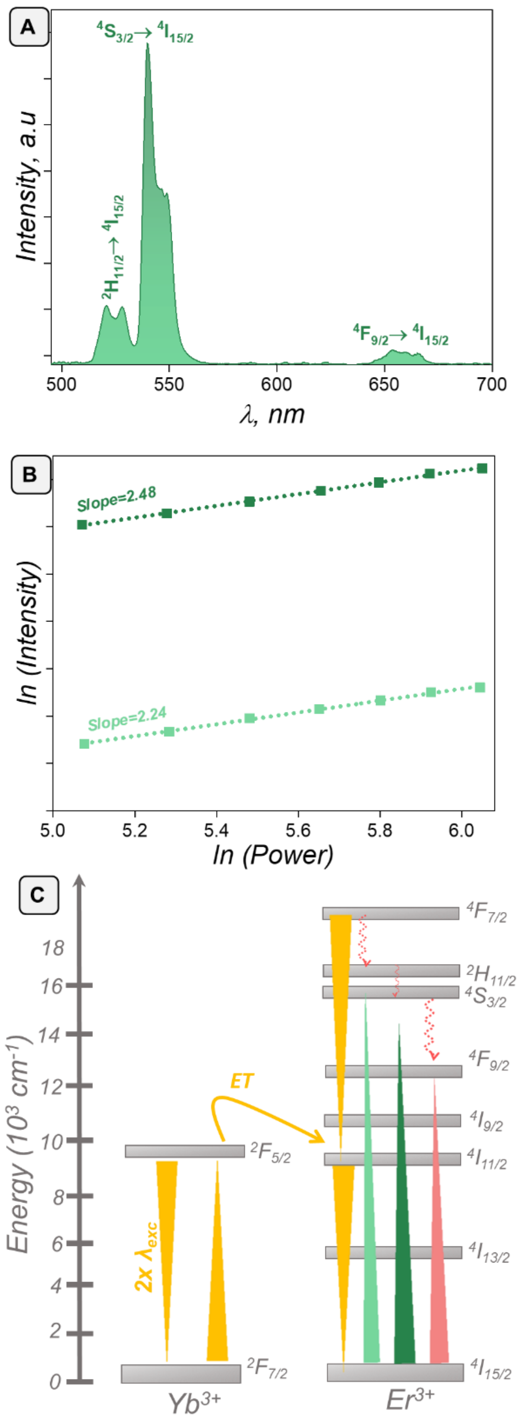

Fig. S10. Upconversion within Er, Yb doped $NaYF_4$ particles under 980 nm excitation. (a) Upconversion spectra, (b) number of photons involved in upconversion and (c) upconversion mechanism of Er^{3+}, Yb^{3+} codoped $NaYF_4$ under near infrared 980 nm excitation wavelength.

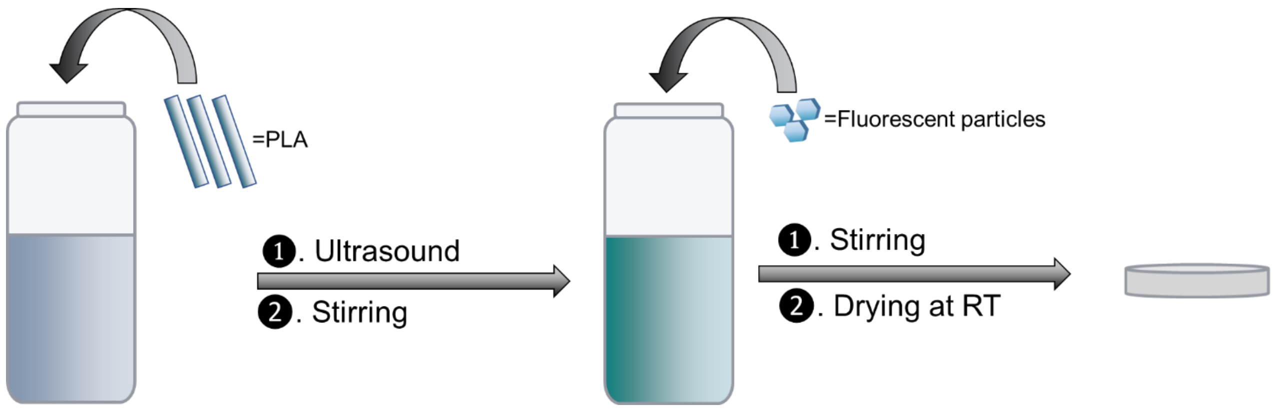

Fig. S11. Preparing fluorescent composites. Preparation of fluorescent composites using polylactic acid (PLA) polymer as matrix via solvent evaporation process.

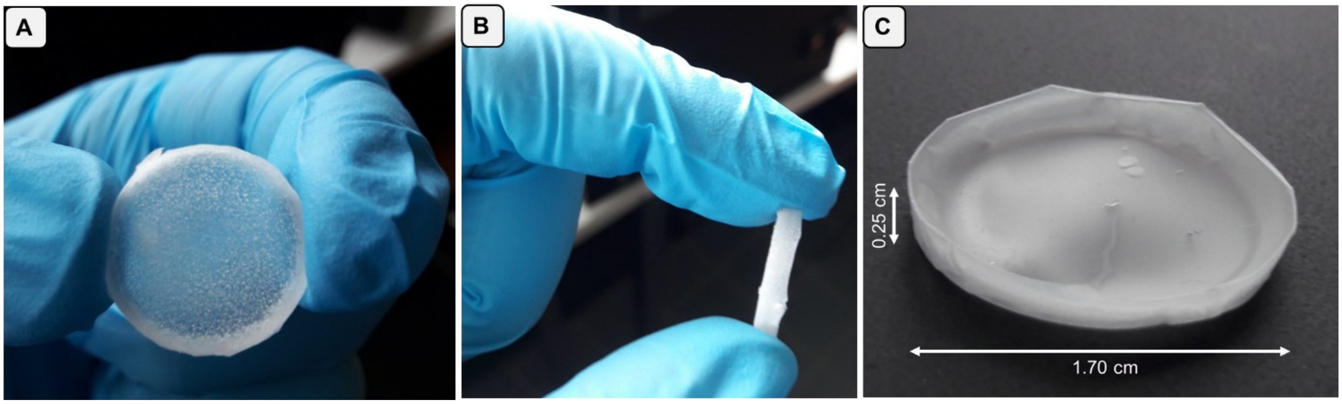

Fig. S12. Digital images of fluorescent composites. Different images of fluorescent composites prepared via the solvent evaporation process: (a) front view, (b) side view, and (c) dimensions of the composite.

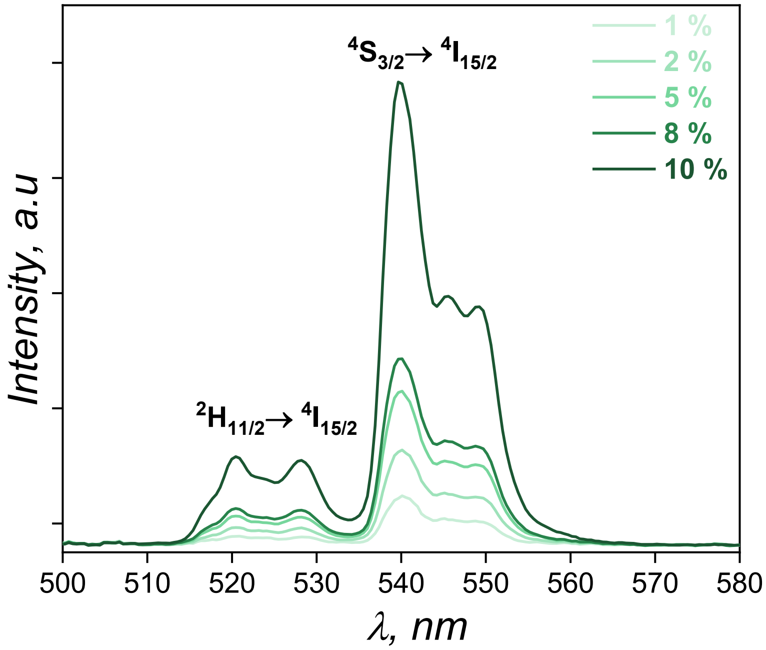

Fig. S13. Optimizing the concentration of green emitting particles within polymer matrix. Photoluminescence of the green emitting particles within the polylactic acid as a function of the concentration. The excitation wavelength is 980 nm.

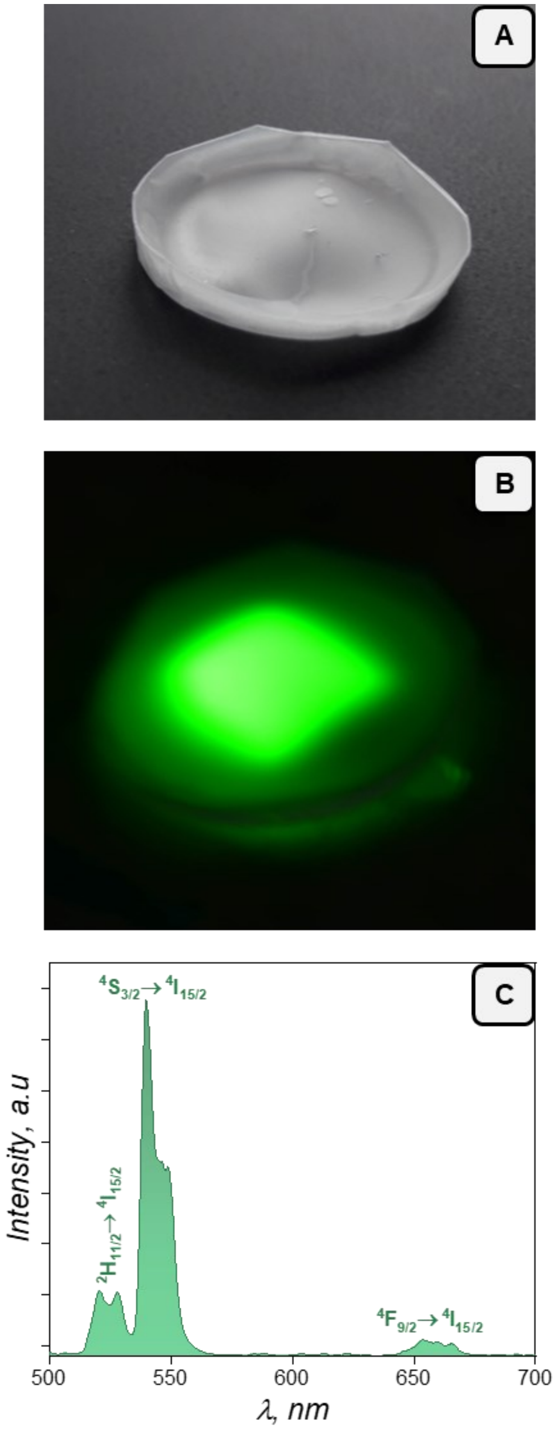

Fig. S14. Fluorescent composites under 980 nm excitation. Digital image of the green emitting composites, embedded into biodegradable polylactic acid matrix, under: (a) daylight, (b) under NIR 980 nm, and (c) recorded photoluminescence spectra.

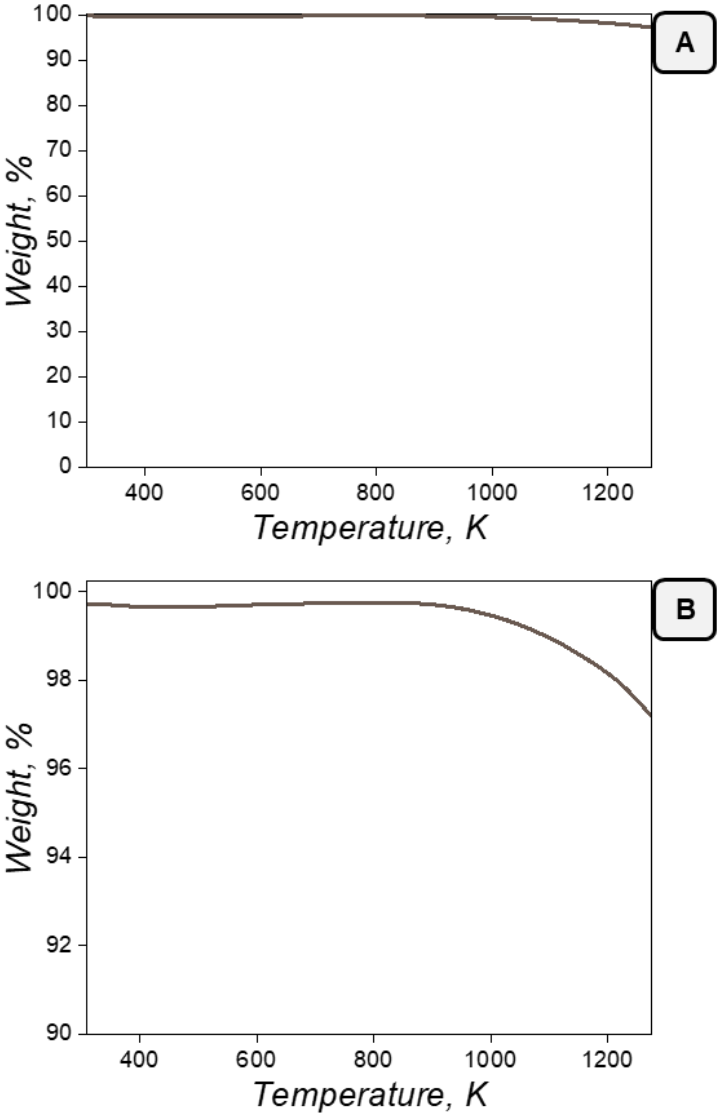

Fig. S15. Thermal stability of green emitting particles. Thermogravimetric analysis of the lanthanide doped materials: (a) full weight range, and (b) weight range limited to 90% to highlight the loss.

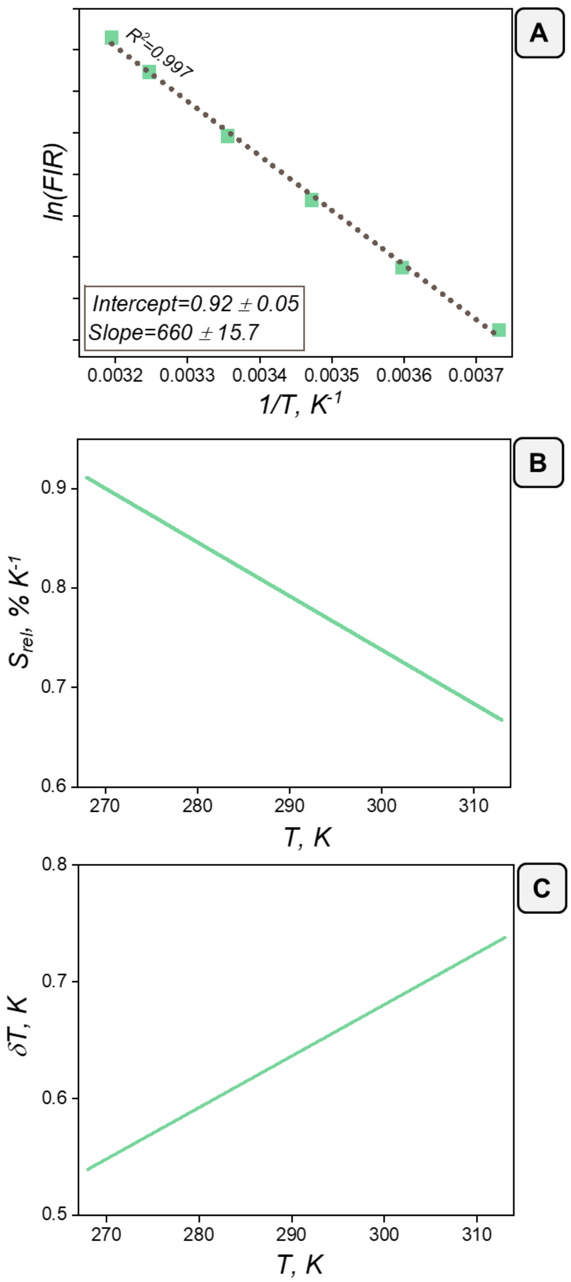

Fig. S16. Thermometric performance of I-SeedSam. (a) Logarithmic form of FIR as a function of temperature for the determination of the slope and intercept. (b) Relative thermal sensitivity (S_{rel}), and (c) the temperature resolution (δT) of the fluorescent *Acer campestre* seeds as a function of temperature.

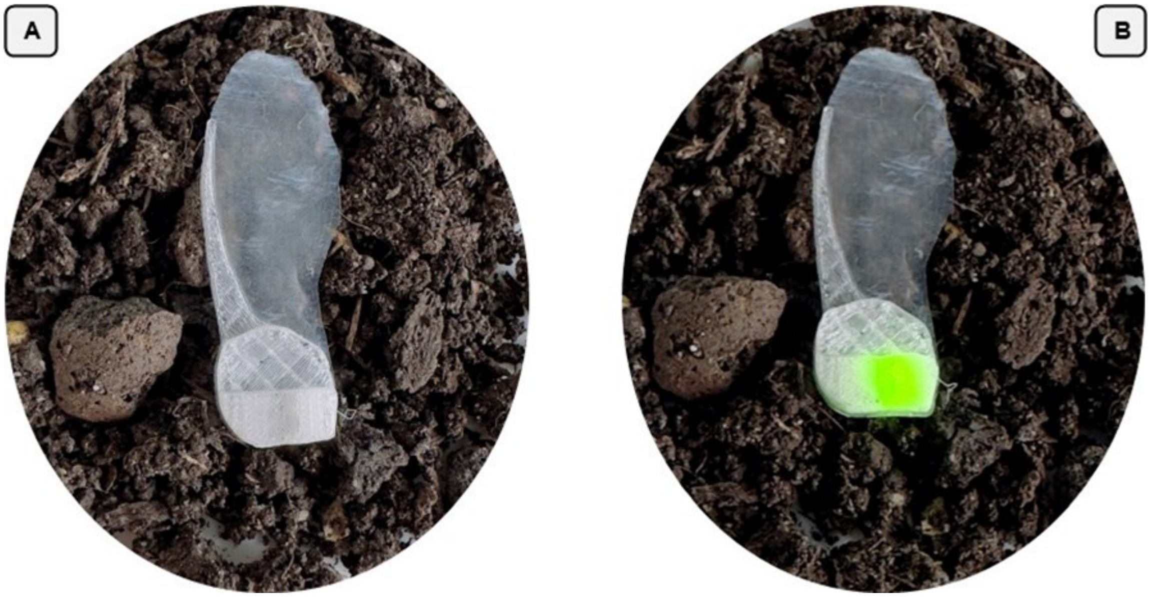

Fig. S17. Monitoring the temperature of the topsoil with I-SeedSam. Fluorescent I-SeedSam on topsoil under (a) daylight, and (b) daylight and excited with a 980 nm wavelength laser pointer.

Movie S1.
Free fall of a natural *Acer campestre* seed (top view).

Movie S2.
Free fall of a natural *Acer campestre* seed (lateral view).

Movie S3.
Free fall of an artificial *Acer campestre* seed (bottom view).

Movie S4.
Free fall of an artificial *Acer campestre* seed (lateral view).

Movie S5.
Outdoors dispersal experiments for the natural and artificial *Acer campestre* seeds.

Movie S6.
Drone I-SeedSam release test.

Movie S7.
Production of fluorescent filaments via an extruder.

Movie S8.
Collecting fluorescent filament for 3D printing.

REFERENCES AND NOTES

1. R. Ouni, K. Saleem, Framework for sustainable wireless sensor network based environmental monitoring. *Sustainability* **14**, 8356 (2022).

2. L. Cecchini, S. Mariani, M. Ronzan, A. Mondini, N. M. Pugno, B. Mazzolai, 4D printing of humidity-driven seed inspired soft robots. *Adv. Sci.* **10**, 2205146 (2023).

3. D. Luo, A. Maheshwari, A. Danielescu, J. Li, Y. Yang, Y. Tao, L. Sun, D. K. Patel, G. Wang, S. Yang, T. Zhang, L. Yao, Autonomous self-burying seed carriers for aerial seeding. *Nature* **614**, 463–470 (2023).

4. M. Seale, N. Nakayama, From passive to informed: Mechanical mechanisms of seed dispersal. *New Phytol.* **225**, 653–658 (2020).

5. V. Iyer, H. Gaensbauer, T. L. Daniel, S. Gollakota, Wind dispersal of battery-free wireless devices. *Nature* **603**, 427–433 (2022).

6. B. H. Kim, K. Li, J. T. Kim, Y. Park, H. Jang, X. Wang, Z. Xie, S. M. Won, H. J. Yoon, G. Lee, W. J. Jang, K. H. Lee, T. S. Chung, Y. H. Jung, S. Y. Heo, Y. Lee, J. Kim, T. Cai, Y. Kim, P. Prasopsukh, Y. Yu, X. Yu, R. Avila, H. Luan, H. Song, F. Zhu, Y. Zhao, L. Chen, S. H. Han, J. Kim, S. J. Oh, H. Lee, C. H. Lee, Y. Huang, L. P. Chamorro, Y. Zhang, J. A. Rogers, Three-dimensional electronic microfliers inspired by wind-dispersed seeds. *Nature* **597**, 503–510 (2021).

7. H. J. Yoon, G. Lee, J. T. Kim, J. Y. Yoo, H. Luan, S. Cheng, S. Kang, H. L. T. Huynh, H. Kim, J. Park, J. Kim, S. S. Kwak, H. Ryu, J. Kim, Y. S. Choi, H. Y. Ahn, J. Choi, S. Oh, Y. H. Jung, M. Park, W. Bai, Y. Huang, L. P. Chamorro, Y. Park, J. A. Rogers, Biodegradable, three-dimensional colorimetric fliers for environmental monitoring. *Sci. Adv.* **8**, eade3201 (2022).

8. F. Wiesemüller, Z. Meng, Y. Hu, A. Farinha, Y. Govdeli, P. H. Nguyen, G. Nyström, M. Kovač, Transient bio-inspired gliders with embodied humidity responsive actuators for environmental sensing. *Front. Robot. AI* **9**, 1011793 (2022).

9. Z. Wang, B. Zhang, D. Guan, Take responsibility for electronic-waste disposal. *Nature* **536**, 23–25 (2016).

10. K. Yasuda, A. Azuma, The autorotation boundary in the flight of samaras. *J. Theor. Biol.* **185**, 313–320 (1997).

11. R. A. Norberg Autorotation, self-stability, and structure of single-winged fruits and seeds (samaras) with comparative remark on animal flight. *Biol. Rev.* **48**, 561–596 (1973).

12. S. J. Lee, E. J. Lee, M. H. Sohn, Mechanism of autorotation flight of maple samaras (*Acer palmatum*). *Exp. Fluids* **55**, 1718 (2014).

13. D. Lentink, W. B. Dickson, J. L. van Leeuwen, M. H. Dickinson, Leading-edge vortices elevate lift of autorotating plant seeds. *Science* **324**, 1438–1440 (2009).

14. B. Mazzolai, S. Mariani, M. Ronzan, L. Cecchini, I. Fiorello, K. Cikalleshi, L. Margheri, Morphological computation in plant seeds for a new generation of self-burial and flying soft robots. *Front. Robot. AI* **8**, 797556 (2021).

15. S. K. H. Win, L. S. T. Win, D. Sufiyan, G. S. Soh, S. Foong, An agile samara-inspired single-actuator aerial robot capable of autorotation and diving. *IEEE Trans. Robot.* **38**, 1033–1046 (2022).

16. P. Pounds, S. Singh, Samara: Biologically inspired self-deploying sensor networks. *IEEE Potentials* **34**, 10–14 (2015).

17. F. Wiesemüller, A. Miriyev, M. Kovac, Zero-footprint eco-robotics: A new perspective on biodegradable robots, in *2021 Aerial Robotic Systems Physically Interacting with the Environment* (*AIRPHARO*) (2021), pp. 1–6.

18. S. S. Sethi, M. Kovac, F. Wiesemüller, A. Miriyev, C. M. Boutry, Biodegradable sensors are ready to transform autonomous ecological monitoring. *Nat. Ecol. Evol.* **6**, 1245–1247 (2022).

19. F. Wiesemüller, S. Meyer, Y. Hu, D. Bachmann, A. Parrilli, G. Nyström, M. Kovač, Biopolymer cryogels for transient ecology-drones. *Adv. Intell. Syst.* **5**, 2300037 (2023).

20. I. Cooperstein, E. Sachyani-Keneth, E. Shukrun-Farrell, T. Rosental, X. Wang, A. Kamyshny, S. Magdassi, Hybrid materials for functional 3D printing. *Adv. Mater. Interfaces* **5**, 1800996 (2018).

21. G. Palmara, F. Frascella, I. Roppolo, A. Chiappone, A. Chiadò, Functional 3D printing: Approaches and bioapplications. *Biosens. Bioelectron.* **175**, 112849 (2021).

22. C. D. S. Brites, P. P. Lima, N. J. O. Silva, A. Millán, V. S. Amaral, F. Palacio, L. D. Carlos, Thermometry at the nanoscale. *Nanoscale* **4**, 4799–4829 (2012).

23. A. Nexha, J. J. Carvajal, M. C. Pujol, F. Díaz, M. Aguiló, Lanthanide doped luminescence nanothermometers in the biological windows: Strategies and applications. *Nanoscale* **13**, 7913–7987 (2021).

24. J. Z. Gul, M. Sajid, M. M. Rehman, G. U. Siddiqui, I. Shah, K. H. Kim, J. W. Lee, K. H. Choi, 3D printing for soft robotics - A review. *Sci. Technol. Adv. Mater.* **19**, 243–262 (2018).

25. S. Farah, D. G. Anderson, R. Langer, Physical and mechanical properties of PLA, and their functions in widespread applications - A comprehensive review. *Adv. Drug Deliv. Rev.* **107**, 367–392 (2016).

26. D. Garlotta, A literature review of poly(lactic acid). *J. Polym. Environ.* **9**, 63–84 (2001).

27. A. Chamas, H. Moon, J. Zheng, Y. Qiu, T. Tabassum, J. H. Jang, M. Abu-Omar, S. L. Scott, S. Suh, Degradation rates of plastics in the environment. *ACS Sustain. Chem. Eng.* **8**, 3494–3511 (2020).

28. S. M. Satti, A. A. Shah, T. L. Marsh, R. Auras, Biodegradation of poly(lactic acid) in soil microcosms at ambient temperature: Evaluation of natural attenuation, bio-augmentation and bio-stimulation. *J. Polym. Environ.* **26**, 3848–3857 (2018).

29. G. Chen, H. Qiu, P. N. Prasad, X. Chen, Upconversion nanoparticles: Design, nanochemistry, and applications in theranostics. *Chem. Rev.* **114**, 5161–5214 (2014).

30. V. I. Sokolov, A. V. Zvyagin, S. M. Igumnov, S. I. Molchanova, M. M. Nazarov, A. V. Nechaev, A. G. Savelyev, A. A. Tyutyunov, E. V. Khaydukov, V. Y. Panchenko, Determination of the refractive index of β-$NaYF_4/Yb^{3+}/Er^{3+}/Tm^{3+}$ nanocrystals using spectroscopic refractometry. *Opt. Spectrosc.* **118**, 609–613 (2015).

31. R. Auras, B. Harte, S. Selke, An overview of polylactides as packaging materials. *Macromol. Biosci.* **4**, 835–864 (2004).

32. G. K. Nave, Jr., N. Hall, K. Somers, B. Davis, H. Gruszewski, C. Powers, M. Collver, D. G. Schmale, III, S. D. Ross, Wind dispersal of natural and biomimetic maple samaras. *Biomimetics* **6**, 23 (2021).

33. H. Takahashi, T. Yamauchi, T. Colmer, M. Nakazono, Aerenchyma formation in plants, in *Low-Oxygen Stress in Plants*, J. T. van Dongen, F. Licausi, Eds. (Springer, Vienna, 2014), vol. 21, pp. 247–265.

34. B. Albinsson, S. Li, K. Lundquist, R. Stomberg, The origin of lignin fluorescence. *J. Mol. Struct.* **508**, 19–27 (1999).

35. D. S. Green, The terminal velocity and dispersal of spinning samaras. *Am. J. Bot.* **67**, 1218–1224 (1980).

36. "Prusa i3 MK3S"; www.prusa3d.com/product/original-prusa-i3-mk3s-3d-printer-3/#specs.

37. D. F. Greene, E. A. Johnson, A model of wind dispersal of winged or plumed seeds. *Ecology* **70**, 339–347 (1989).

38. A. Okubo, S. A. Levin, A theoretical framework for data analysis of wind dispersal of seeds and pollen. *Ecology* **70**, 329–338 (1989).

39. Z. Duan, Y. Li, J. Wang, G. Zhao, S. Svanberg, Aquatic environment monitoring using a drone-based fluorosensor. *Appl. Phys. B* **125**, 108 (2019).

40. C. Dong, Remote sensing, hydrological modeling and in situ observations in snow cover research: A review. *J. Hydrol.* **561**, 573–583 (2018).

41. F. Vetrone, R. Naccache, A. Zamarrón, A. Juarranz de la Fuente, F. Sanz-Rodríguez, L. Martinez Maestro, E. Martín Rodriguez, D. Jaque, J. García Solé, J. A. Capobianco, Temperature sensing using fluorescent nanothermometers. *ACS Nano* **4**, 3254–3258 (2010).

42. K. W. Krämer, D. Biner, G. Frei, H. U. Güdel, M. P. Hehlen, S. R. Lüthi, Hexagonal sodium yttrium fluoride based green and blue emitting upconversion phosphors. *Chem. Mater.* **16**, 1244–1251 (2004).

43. A. D. Pickel, A. Teitelboim, E. M. Chan, N. J. Borys, P. J. Schuck, C. Dames, Apparent self-heating of individual upconverting nanoparticle thermometers. *Nat. Commun.* **9**, 4907 (2018).

44. H. Suo, X. Zhao, Z. Zhang, Y. Wang, J. Sun, M. Jin, C. Guo, Rational design of ratiometric luminescence thermometry based on thermally coupled levels for bioapplications. *Laser Photon. Rev.* **15**, 2000319 (2021).

45. C. D. S. Brites, A. Millán, L. D. Carlos, Lanthanides in luminescent thermometry, in *Handbook on the Physics and Chemistry of Rare Earths*, B. Jean-Claude, P. K. Vitalij, Eds. (Elsevier, 2016), vol. **49**, pp. 339–427.

46. A. Nexha, M. C. Pujol, F. Díaz, M. Aguiló, J. J. Carvajal, Luminescence nanothermometry using self-assembled Er^{3+}, Yb^{3+} doped Y_2O_3 nanodiscs: Might the upconversion mechanism condition their use as primary thermometers? *Opt. Mater.* **134**, 113216 (2022).

47. A. A. Singh, M. E. Genovese, G. Mancini, L. Marini, A. Athanassiou, Green processing route for polylactic acid-cellulose fiber biocomposites. *ACS Sustain. Chem. Eng.* **8**, 4128–4136 (2020).

48. S. W. Pattinson, A. J. Hart, Additive manufacturing of cellulosic materials with robust mechanics and antimicrobial functionality. *Adv. Mater. Technol.* **2**, 1600084 (2017).

49. K. Cikalleshi, S. Mariani, B. Mazzolai, in *Biomimetic and Biohybrid Systems*, F. Meder, A. Hunt, L. Margheri, A. Mura, B. Mazzolai, Eds. (Springer Nature Switzerland, 2023), pp. 117–129.

50. B. Mazzolai et al., in *GoodIT '22: Proceedings of the 2022 ACM Conference on Information Technology for Social Good*. (ACM Digital Library, 2022), pp. 265–268.

51. T. Hamann, E. Smets, F. Lens, A comparison of paraffin and resin-based techniques used in bark anatomy. *Taxon* **60**, 841–851 (2011).

52. C. A. Schneider, W. S. Rasband, K. W. Eliceiri, NIH image to ImageJ: 25 years of image analysis. *Nat. Methods* **9**, 671–675 (2012).

53. S. Valdes, I. Urza, P. E. I. Pounds, S. Singh, Samara: Low-cost deployment for environmental sensing using passive autorotation, in *Robotics: Science and Systems Workshop on Robotics for Environmental Monitoring (Citeseer)*, Sydney, Australia, July 2012.
